# Optimizing irrigation and fertilization management enhances alfalfa seed yield components through improved soil nutrient availability and leaf photosynthetic efficiency

**DOI:** 10.3389/fpls.2025.1655710

**Published:** 2025-08-29

**Authors:** Jinfeng Hui, Yanliang Sun, Kongqin Wei, Andrew D. Cartmill, Ignacio F. López, Chunhui Ma, Qianbing Zhang

**Affiliations:** ^1^ College of Animal Science and Technology, Shihezi University, Shihezi, Xinjiang, China; ^2^ School of Agriculture and Environment, Massey University, Palmerston North, New Zealand

**Keywords:** drip irrigation optimization, alfalfa seed production, seed morphology, soil nutrient availability, photosynthetic efficiency

## Abstract

**Introduction:**

Addressing the challenges of inefficient water-fertilizer utilization and suboptimal seed yield in alfalfa (*Medicago sativa* L.) seed production systems, we investigated the effects of differential irrigation-fertilization regimes on soil nutrient dynamics, photosynthetic performance, and yield parameters. This study aims to optimize seed production while elucidating the response mechanisms linking soil nutrient availability, foliar photosynthetic efficiency, and seed yield outcomes. This experiment employed drip irrigation to address production constraints in alfalfa seed cultivation.

**Methods:**

Using ‘WL354HQ’ and ‘Xinmu No.4’ as the experimental materials, a two-factor randomized block design was adopted, with three fertilization levels: F_0_ (no fertilizer), F_1_ (90 kg·ha^-1^ N 75 kg·ha^-1^ P_2_O_5_, 12 kg·ha^-1^ K_2_O), and F_2_ (120 kg·ha^-1^ N, 100 kg·ha^-1^ P_2_O_5_, 15 kg·ha^-1^ K_2_O), and combined with three irrigation levels W_1_ (1650 m^3^·ha^-1^), W_2_ (2500 m^3^·ha^-1^), and W_3_ (3350 m^3^·ha^-1^).

**Results:**

Water and fertilizer management is a prerequisite for high yield of alfalfa seeds, and the impact of fertilization on seed yield is greater than that of irrigation. Compared to the non-fertilized control (F_0_W_1_), the F_2_W_2_ treatment significantly increased soil nutrients in the 0–20 cm layer: soil total nitrogen content (+52.17%), total phosphorus content (+18.72%), and organic carbon content (+16.85%), and available phosphorus content (+37.34%), and alkali-hydrolyzable nitrogen content (+17.45%). Notably, F_2_W_2_ enhanced net photosynthetic rate by 35.04% despite reduced stomatal conductance (-2.14%) and intercellular CO_2_ concentration (-9.50%), thereby promoting assimilate partitioning to reproductive organs. Consequently, seed dimensional parameters (width: +53.02%; thickness: +21.75%) and germination rate (+23.11%) were significantly improved (*P* < 0.05), increasing the seed yields of WL354HQ and Xinmu No.4 by 42.76% and 49.81% respectively. Correlation analysis revealed significant (*P* < 0.01) positive associations between seed yield and seed length, seed width, seed thickness, chlorophyll a, carotenoids, total chlorophyll content, and net photosynthetic rate. Principal component analysis showed that the optimal fertilization level was N 120 kg·ha^-1^; P_2_O_5–_100 kg·ha^-1^; K_2_O 15 kg·ha^-1^, with an irrigation level of 2500 m^3^·ha^-1^ (F_2_W_2_) as the optimal model.

**Discussion:**

This optimized model significantly enhanced alfalfa seed yield formation, photosynthetic characteristics, and soil nutrient availability, which provided a theoretical basis for high yield cultivation of alfalfa seed production in arid areas.

## 1 Introduction

Alfalfa (*Medicago sativa* L.), a globally cultivated leguminous species, produces high forage and hay yields with excellent nutritional quality and high preference (Mu et al., 2025). Recent optimization of China’s forage cropping structure and grassland improvement efforts, coupled with expanded cultivation of exotic and non-naturalized forage species, have increased market demand for high-quality, high-yielding alfalfa seeds (Li et al., 2023a). In 2024, domestic alfalfa seed demand reached approximately 10,000 tons, yet domestic production supplied only 2,700 tons, indicating significant reliance on imports to satisfy this growing demand (Liu et al., 2024). This escalating supply-demand imbalance necessitates re-evaluating seed production strategies, particularly emphasizing precise water and fertilizer management. While advancements have been made in alfalfa water and fertilizer management—including water-nitrogen strategies (Lv et al., 2025), such as water monitoring based on unmanned aerial vehicles (Wu et al., 2025) and optimized irrigation systems for saline water (Lou et al., 2025), there are still research gaps: Specifically, under subsurface drip irrigation (SDI) conditions, the synergistic effects of fertilizer application and irrigation volume remain unquantified; there is a lack of verification of the optimization of nitrogen, phosphorus, and potassium fertilizer ratios, and the physiological mechanisms between soil nutrients and seed yield have not been fully elucidated.

Alfalfa seed production is highly dependent on water availability, as any water stress at any growth stage can negatively affect growth and yield (Montazar and Sadeghi, 2008). Photosynthetic activity in plants is the foundation for dry matter accumulation and yield formation, under dry conditions, irrigation stimulates plant growth through active photosynthetic activity, facilitating nutrient accumulation and high annual yield (Eltarabily et al., 2024). However, while high irrigation (> 3000 m3·ha-1) during alfalfa’s initial flowering period may increase inflorescence number, which may reduce pollen viability by 38.2% and nectar concentration by 22.7%, resulting in a 16.5-24.3% decrease in podding rate (Wang et al., 2025; Zhang and Guo, 2025). Furthermore, high irrigation rates may also increase nitrate leaching, elevating subsoil (40–60 cm) NO_3_⁻-N accumulation by 22.6-34.8%, while compromising soil aggregate stability (Liu et al., 2018). In contrast, water deficit during alfalfa branching has been reported to enhance shoot appearance capacity and improve lodging resistance. However, it has been reported that under these conditions plant height, stem diameter, and thousand-seed weight decrease, probably reflecting an impaired nutrient translocation as a result of the water deficit (Gao et al., 2023).

Optimizing the ratio of nitrogen (N), phosphorus (P), and potassium (K) fertilizers for the growth requirements of seed alfalfa may improve the energy efficiency of seed production (Jia et al., 2024). Studies have shown that the application of N fertilizer (120–150 kg·ha^-1^) can increase the activity of glutamine synthase by 42.7% and drive the expansion of N reservoir capacity (Ou et al., 2021). It has been reported that this metabolic adjustment promotes phloem-mediated N remobilization via sucrose-amino acid cotransport systems, resulting in 23.05% seed yield enhancement (Lu et al., 2019). However, when the N application rate (the N was as ammonium form), exceeded 180 kg·ha^-1^, the pH value of the rhizosphere decreased by 0.8-1.2, resulting in a 57.3% reduction in the abundance of Sinorhizobium meliloti, a N-fixing bacterium which forms symbiotic relationships with the roots of leguminous plants. This disrupted the structure of the soil microbial community, leading to a decrease in yield and fertilizer waste/loss (Wei et al., 2025). Parallel investigations in wheat (*Triticum aestivum* L.) demonstrated that P-mediated PEPC activation increases fertile florets per inflorescence by 26.3% (Lollato et al., 2019). In addition, the combined application of N and P can increase the leaf area of alfalfa and enhance the net photosynthetic rate of leaves (Sun et al., 2022). Furthermore, precision nutrient management has also been reported to modulate soil properties through pH buffering and enhance soil organic matter content and aggregate stability (Song et al., 2025).

This study examined the influence of varied irrigation-fertilization regimes on soil nutrient dynamics, photosynthetic performance, and yield formation parameters in alfalfa, aiming to optimize seed production. Critically, within a drip-irrigation alfalfa seed production system in an arid region, we quantified the water-fertilizer synergy threshold (N-P-K × irrigation amount) for the first time. This threshold elucidates the response mechanisms linking soil nutrient availability, photosynthetic efficiency, and seed yield. Therefore, we hypothesize that: (1) Under drip irrigation in an arid region, the synergistic application of N, P, and K fertilizers combined with moderate irrigation (< 3000 m3·ha-1) will significantly enhance alfalfa seed yield components by optimizing soil nutrient availability and photosynthetic carbon assimilation efficiency, and (2) optimized irrigation-fertilization management minimizes soil nitrogen leaching while enhancing N retention efficiency, thereby creating a rhizosphere nutrient buffer that sustains photosynthetic competence under moderate water deficit. This synergy ensures continuous carbon assimilation for reproductive sink development. Consequently, this study quantified response thresholds relevant to conventional practices and established boundaries for maintaining productivity without resource overuse, providing a theoretical basis for improving irrigation and fertilizer management in alfalfa seed production.

## 2 Materials and methods

### 2.1 Experimental site

**Figure 1:**
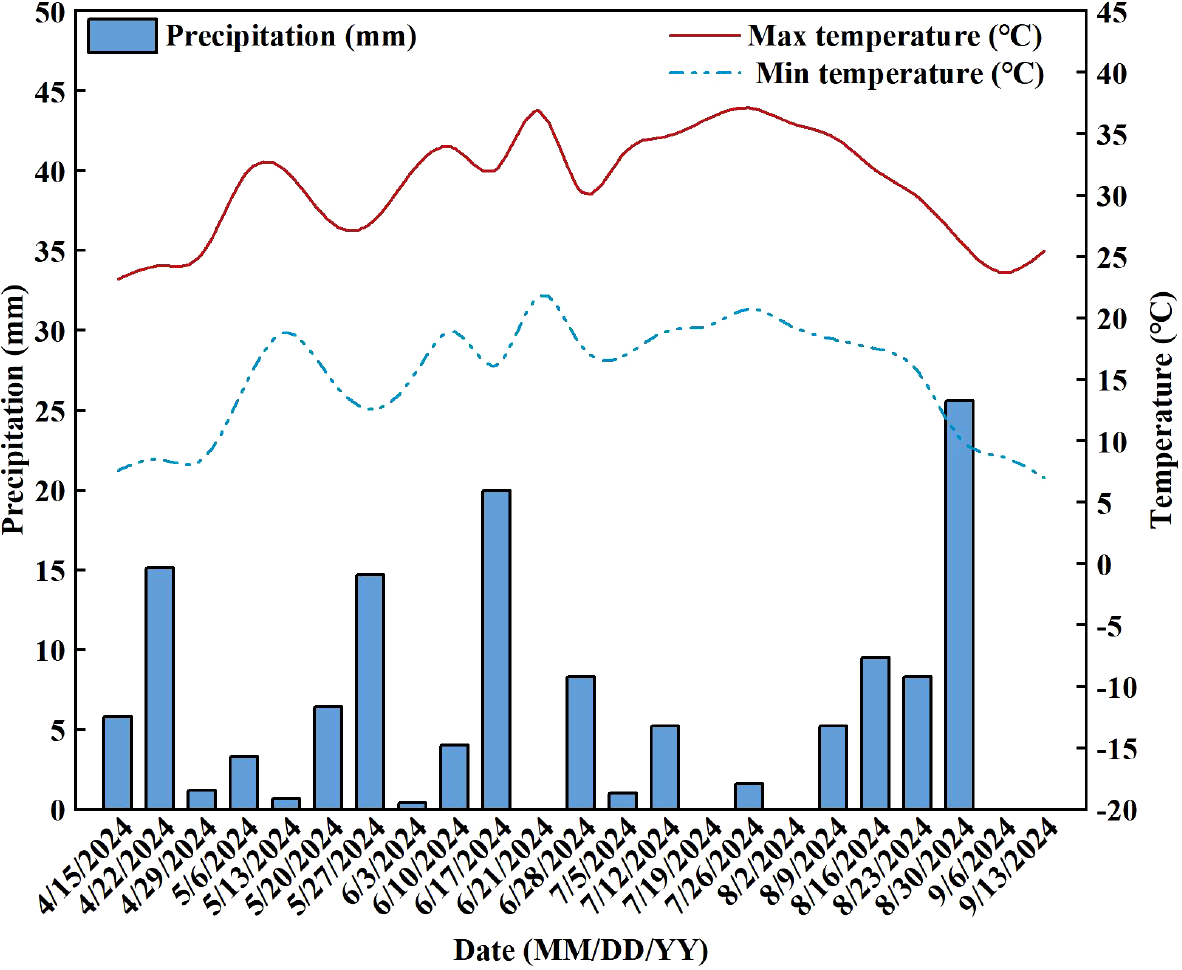
Temperature and precipitation during the growth season of alfalfa in 2024.

The study was carried out in the forage experimental station of Shihezi University in Shihezi City, Xinjiang (44°20’ N, 86°30’ E; altitude 420 m) in 2024. The climate corresponds to a temperate continental climate with arid characteristics (Zhang et al., 2024), with average annual temperature, daylight, rainfall (concentrated late May to late July), and evaporation of 5-10°C, 2721–2818 h, 190–260 mm, and 1100–1400 mm (The temperature and precipitation during the growth season are shown in **Figure 1**), respectively. The soil type of the experimental field was a gray desert soil. The basic nutrient content of tillage soil layer (0–20 cm) was total N 1.08 mg·g^-1^, alkali-hydrolyzable N 124.17 mg·kg^-1^, available P 15.42 mg·kg^-1^, available K 107.90 mg·kg^-1^, organic C 11.32 mg·g^-1^, bulk density 1.63 g·cm^-3^, pH = 7.54. The irrigation water comes from a local underground well (200 m depth), the pH value of the water was measured before the experiment (pH = 7.2), and the same water was used for all treatments to eliminate the variation caused by the water source.

### 2.2 Test materials

Two alfalfa cultivars ‘WL354HQ’ and ‘Xinmu No.4’ (**Table 1**) were selected for use in this study. Xinmu No.4 is a drought-resistant and high-yield alfalfa cultivar bred by Xinjiang Agricultural University(Urumqi, China). and is widely used in the Xinjiang region. Two contrasting cultivars were selected: ‘WL354HQ’ (drought-adapted, US origin) and ‘Xinmu No.4’ (high-yielding Chinese germplasm). This design tests treatment effects across diverse genetic backgrounds, ensuring findings are not genotype-specific. Both cultivars were grown on the same randomized plots to control soil heterogeneity. Fertilizer type: granular calcium superphosphate (P_2_O_5_ = 46%), potassium sulfate (K_2_O = 50%), urea (N = 46%).

**Table 1:** Provenance information of alfalfa cultivars.

Cultivar	Source	Country
WL354HQ	Beijing Rytway Seed Co., Ltd.	United States of America
Xinmu No.4	Xinjiang Agricultural University	China

### 2.3 Experimental design and management

Prior to experimentation, we surveyed water and fertilizer management practices in local high-yield alfalfa seed fields (400 ± 30 kg·ha⁻¹). The fertilizer application amount were N 120–150 kg·ha^-1^, P_2_O_5_ 100–120 kg·ha^-1^, and K_2_O 15–20 kg·ha^-1^, with irrigation volumes of 2500–3500 m^3^·ha^-1^ was the common practice in the local high-yield alfalfa seed farms. Based on the soil physical and chemical experiment results of the experimental field and the previous research results of the research group (Sun et al., 2022; Zhao et al., 2022) and the planting cost, the final application rates of nitrogen, phosphorus, and potassium fertilizers and the irrigation volume for this experiment were determined, the fertilizer level is the local minimum and 25% lower than the minimum, and the irrigation level is adjusted to ±33% of the local minimum, these application amounts were adjusted by reducing by 25% to determine the fertilization level, and adjust these irrigation rates by increasing and decreasing by 33% to determine the appropriate irrigation level. This study used a two-factor randomized block design, with three fertilization levels: F_0_: no fertilization; F_1_: 90 kg·ha^-1^ N, 75 kg·ha^-1^ P_2_O_5_, 12 kg·ha^-1^ K_2_O; F_2_: 120 kg·ha^-1^ N, 100 kg·ha^-1^ P_2_O_5_, and 15 kg·ha^-1^ K_2_O, and three irrigation levels: W_1_: 1650 m^3^·ha^-1^ (low irrigation); W_2_: 2500 m^3^·ha^-1^ (medium irrigation); and W_3_: 3350 m^3^·ha^-1^ (high irrigation), for a total of with three blocks, giving a total of 27 experimental plots.

Plots 12 m^2^ (3 m × 4 m) with 1 m wide vegetated protective perimeter strip were established (April 15, 2024). Alfalfa seeds were sown at 2 cm depth, on 20 cm centers (20 cm row spacing and 20 cm plant spacing within row). Before sowing, subsurface drip irrigation was buried at 10 cm depth, laid parallel to the alfalfa row every 80 cm, with ‘drippers’ evenly distributed at 20 cm intervals. After sowing, to encourage seed germination, plots were brought to field capacity through the application of 0.6 m^3^ of water.

Water and fertilizer treatment commenced on May 27, 2024 (**Table 2**). Throughout the experimental period, irrigation scheduling was dynamically adjusted in real-time based on soil moisture monitoring and precipitation data. A total of seven irrigation events and three fertilizer applications were implemented, with fertilization administered concurrently through the irrigation system. All other standard agronomic practices were uniformly maintained across treatments.

**Table 2:** Fertilization and irrigation schemes.

Treatment^1^	Irrigation amount (m^3^·ha^-1^)							Fertilizer application amount N-P_2_O_5_-K_2_O (kg·ha^-1^)		
	05-27^2^	06-15	06-30	07-14	07-31	08-15	08-28	06-15	07-14	08-15
F_0_W_1_	300	300	300	200	200	200	150	0-0-0	0-0-0	0-0-0
F_0_W_2_	400	400	400	350	350	350	250	0-0-0	0-0-0	0-0-0
F_0_W_3_	500	500	500	500	500	500	350	0-0-0	0-0-0	0-0-0
F_1_W_1_	300	300	300	200	200	200	150	59(30-25-4)	59(30-25-4)	59(30-25-4)
F_1_W_2_	400	400	400	350	350	350	250	59(30-25-4)	59(30-25-4)	59(30-25-4)
F_1_W_3_	500	500	500	500	500	500	350	59(30-25-4)	59(30-25-4)	59(30-25-4)
F_2_W_1_	300	300	300	200	200	200	150	70(40-30-5)	80(40-40-5)	70(40-30-5)
F_2_W_2_	400	400	400	350	350	350	250	70(40-30-5)	80(40-40-5)	70(40-30-5)
F_2_W_3_	500	500	500	500	500	500	350	70(40-30-5)	80(40-40-5)	70(40-30-5)

^1^F_0_ denotes no fertilization, F_1_ denotes N 90 kg·ha^-1^; P_2_O_5–_75 kg·ha^-1^; K_2_O 12 kg·ha^-1^, F_2_ denotes N 120 kg·ha^-1^; P_2_O_5–_100 kg·ha^-1^; K_2_O 15 kg·ha^-1^. W_1_ denotes 1650 m^3^·ha^-1^; W_2_ denotes 2500 m^3^·ha^-1^; W_3_ denotes 3350 m^3^·ha^-1^.

^2^Irrigation and fertilizer date.

### 2.4 Physiological and morphological measurements

#### 2.4.1 Determination of physical and chemical properties of soil

Before alfalfa seed were harvested, soil samples were collected (September 14, 2024) from each plot using a 5 - point sampling method (Triberti et al., 2016) to a depth of 60 cm. Briefly, soil samples (cores) were then split into three distinct depths (0–20 cm, 20–40 cm, and 40–60 cm). For each plot the five samples were bulked by depth and soil organic carbon (SOC) content was quantified using the potassium dichromate (K_2_Cr_2_O_7_) oxidation method with external heating, soil total nitrogen (TN) content was determined by the Kjeldahl digestion method with a FOSS 2300 fully automated nitrogen analyzer (FOSS Analytical, Denmark), and soil alkali-hydrolyzable nitrogen (AN) content was measured via alkaline diffusion, and soil total phosphorus (TP) content was determined through sulfuric-perchloric acid digestion followed by spectrophotometric measurement at 700 nm using a UV-2600 spectrophotometer, and soil available phosphorus (AP) content was extracted with 0.5 M NaHCO_3_ (pH 8.5) (Lu, 2000).

#### 2.4.2 Photosynthetic characterization of alfalfa at podding stage

In the early stage of pod formation, on a sunny and cloudless day, between 11:30 am and 1:00 pm, leaf photosynthetic parameters [net photosynthetic rate (Pn, μmol CO_2_·m^-2^·s^-1^), intercellular CO_2_ concentration (Ci, μmol·mol^-1^), transpiration rate (Tr, mmol H_2_O·m^-2^·s^-1^), and stomatal conductance (Gs, mol·m^-2^·s^-1^)] were measured (n= 6) with a LI-6400/XT portable photosynthesis system (LI-COR Environmental, Lincoln, NE, USA) from the 4^th^ - 5^th^ fully expanded, healthy, and uniform leaf at the top of the main stem. All measurements were conducted under natural light with stabilized photosynthetic photon flux density (PPFD) at 1200 ± 50 μmol·m^-2^·s^-1^, chamber CO_2_ concentration maintained at 400 μmol·mol^-1^, and temperature controlled at 28 ± 2°C.

From the same plants used for photosynthetic measurement in each treatment, the 3^th^ - 4^th^ fully expanded leaves, without mechanical damage and consistent growth, at the top of the main stem were collected. Selected leaves were quickly collected and put into a lvight-shielded aluminum foil bag (to avoid photolysis), and placed in a 4°C portable refrigerator (preservation time ≤ 2 h), and then transported to the laboratory. Leaf pigment content was determined using a 95% ethanol grinding extraction method (Dai et al., 2020). Briefly, 0.2 g fresh leaf was placed in pre-cooled mortar with 5 mL 95% ethanol, and a small amount of calcium carbonate powder (protecting chlorophyll) and quartz sand (assisted grinding) were added, homogenize, and the volume was fixed to 25 mL after filtration. The absorbance values of the extracts at 665 nm, 649 nm, and 470 nm were measured (L-UV90 Ultraviolet-Visible Spectrophotometer, Beijing Leber Taike Instrument Co., Ltd., Beijing, China). The contents of chlorophyll a (Chl a; mg·g^-1^), chlorophyll b (Chl b; mg·g^-1^), and total chlorophyll (Chl a + b; mg·g^-1^) were calculated (Zhao et al., 2022):


$$\begin{array}{c} \text{Chl a}=(13.95\times {\text{A}}_{665}-6.88\times {\text{A}}_{649})\times \text{total amount of extract}(\text{L})\\ \times \text{dilution times}/\text{leaf fresh weight}(\text{g}) \end{array}$$

$$\begin{array}{c} \text{Chl b}=(24.96\times {\text{A}}_{649}-7.32\times {\text{A}}_{665})\times \text{total amount of extract}(\text{L})\\ \times \text{dilution times}/\text{leaf fresh weight}(\text{g}) \end{array}$$

$$\begin{array}{c} \text{Car}=[(1000\times {\text{A}}_{470}-2.05\times \text{Ca}-114.8\times \text{Cb})/245]\\ \times \text{total amount of extract}(\text{L})\\ \times \text{dilution times}/\text{leaf fresh weight}(\text{g}) \end{array}$$

Chl a+b=Chl a+Chl b

#### 2.4.3 Growth index

During pod formation phase, the growth indexes of alfalfa in each plot were measured. Briefly, 20 alfalfa plants per plot were randomly selected and plant height (PH; cm), and main stem thick (St; mm) at 5 cm above the soil level, were measured. The number of primary branches (NPB; No.plant^-1^) at each rhizome and the number of secondary branches (NSB; No.plant^-1^) was also determined.

#### 2.4.4 The constituent factors of alfalfa seed yield and resource use efficiency

To ensure the seed yield of alfalfa, the alfalfa in all plots has never been cut from germination to seed harvest. Therefore, at full-bloom stage, 20 alfalfa plants were randomly selected from each plot, and the number of inflorescences per reproductive branch (IPRB; No.branch^-1^) was determined. In addition, 20 inflorescences per plot were randomly picked, packed into plastic bags, and brought back to the laboratory for determination of small flowers per inflorescence (SFPI; No.inflorescence^-1^).

When 75% of the pods in each plot were brown, 20 pod-bearing inflorescences were randomly selected, and the number of pods per inflorescence (PPI; No.inflorescence^-1^) was counted. In addition, pod inflorescence was picked, placed in plastic bags and brought back to the laboratory to determine seeds per pod (SPP; No.pod^-1^).

The 20 alfalfa plants pre-marked for each plot were manually harvested, and each plant was separately placed in a nylon net bag, and allowed to dry naturally. After drying, plants were threshed and cleaned, and the seed yield per plant was weighed, and the seed yield per hectare (ASY; kg·ha^-1^)was determined. Alfalfa seeds harvested from the same treatment were dried and cleaned, and 6 subsamples of a 1000 clean seeds were randomly selected and weighted to give a thousand-seed weight (TSW; g). Pod setting rate (PR; %) was calculated (number of pods on a single inflorescence/number of florets on a single inflorescence × 100%).

Irrigation water use efficiency (IWUE; kg·m^-3^) and fertilizer partial productivity (PFP; kg·kg^-1^) were determined. Where, IWUE was the economic yield of the crop per unit of irrigation water applied and PFP was ratio of the actual seed yield by total fertilizer application (Ali et al., 2018).


IWUE=actual seed yield/total irrigation input

PFP=actual seed yield/total fertilizer application

#### 2.4.5 seeds quality

The morphological indexes [seed length (SL; mm), width (SW; mm), and thickness (ST; mm)] of alfalfa seeds after harvest were measured (vernier caliper; precision 0.01 mm) from 30 seeds total per treatment (10 seed per biological replicate; n = 3). Seed length, width, and thickness were defined as, the maximum longitudinal geometric distance of the seed, the maximum transverse geometric distance of the seed, and the maximum vertical distance between the ventral surface and the dorsal surface, respectively.

For determination of germination characteristics, 200 seeds were randomly selected from each treatment after harvest, disinfected with 95% ethanol for 1 min, rinsed 5 times with distilled water, and evenly placed in a 90 mm glass culture dish between 2 layers of qualitative filter paper (50 seeds per dish; n = 4). Distilled water (4 ml) was added to the filter paper, and the culture dish were moved to a incubator set at 20°C, with 16 hours of light/8 hours of darkness. During the incubation period, 1 ml of distilled water was supplemented daily to keep the the filter paper moist. The number of germinated seeds was determined daily and recorded, with germination defined as the radicle emerging (≥ 2 mm) through the seed coat. Germination potential and rate were determined on the 4^th^ and 10^th^ day, respectively. In addition, 10 seedlings from each treatment group were randomly selected and the length of radicle (RL; cm) and embryo (EL, mm) were measured. Germination potential (GP, %) and germination rate (GR, %) were calculated as follows (Qin et al., 2022):


$$\text{GP}=(\text{the number of seeds germinated on the }4^{\text{th}}\text{day}/\text{the number of seeds tested})\times 100\%$$

$$\text{GR}=(\text{the number of seeds germinated on the }10^{\text{th}}\text{day}/\text{the number of seeds tested})\times 100\%$$

For determination of hard seed rate (HR, %), 150 seeds were randomly selected from each treatment and split into three 50 seed biological replicates (n = 3). Seed were then soaked in distilled water for 48 h under ambient (25 ± 2°C) conditions, and the number of non-imbibed seeds (no sign of water absorption and seed coat not expanded) was determined. The formula for calculating hard seed rate was as follows:


HR=(number of non-imbibibibed seeds/number of test seeds)×100%

### 2.5 Data analysis

Data were sorted and statistically analyzed using Microsoft Excel 2021 (Microsoft Corporation, Redmond, WA, USA), and a two-factor ANOVA was performed with SPSS 20.0 (IBM Corp, Armonk, NY, USA). Additionally, the Duncan method was applied to analyze the effects of fertilizer and irrigation levels on soil physicochemical properties, photosynthetic characteristics, seed composition factors, and seed quality; one-way ANOVA was used to assess the significance (*P* < 0.05) of different irrigation levels under uniform fertilization and different fertilization levels under uniform irrigation on these parameters. Linear fitting and chart generation were conducted using Origin 2021 software (OriginLab, Northampton, MA, USA). Meanwhile, Pearson correlation analysis was employed to examine correlations among the aforementioned indicators, and linear regression was used to fit the relationship between organic C, total N, total P and alfalfa seed yield among different soil layers, as well as the relationship between the input of N, P and K and irrigation volume and seed yield. Moreover, Mantel test was utilized for matrix correlation analysis between seed yield components and resource utilization efficiency, photosynthetic characteristics, and seed quality; principal component analysis was applied to identify the optimal treatment.

## 3 Results

### 3.1 Soil organic carbon, total nitrogen and total phosphorus content

**Figure 2:**
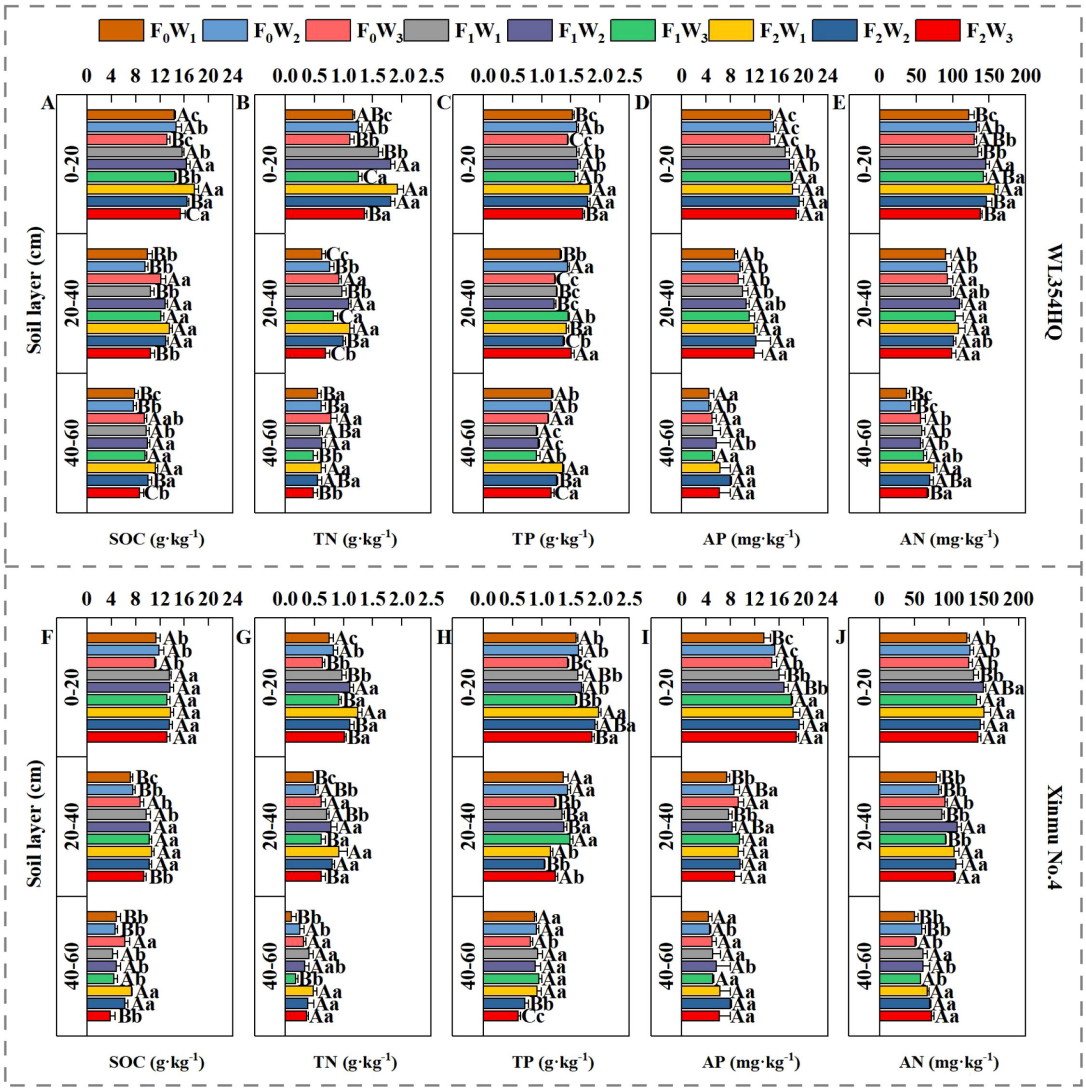
Effects of water and fertilizer management on SOC, TN, TP, AP and AN contents at different soil depths (0–20 cm, 20–40 cm, and 40–60 cm) of alfalfa. **(A–E)** SOC denotes soil organic carbon, TN denotes total nitrogen, TP denotes total phosphorus, AP denotes available phosphorus, and AN denotes alkali-hydrolyzable nitrogen contents for alfalfa cultivar WL354HQ. **(F–J)** SOC denotes soil organic carbon, TN denotes total nitrogen, TP denotes total phosphorus, AP denotes available phosphorus, and AN denotes alkali-hydrolyzable nitrogen contents in for alfalfa cultivar Xinmu No.4. F_0_ denotes no fertilization, F_1_ denotes N 90 kg·ha^-1^; P_2_O_5–_75 kg·ha^-1^; K_2_O 12 kg·ha^-1^, F_2_ denotes N 120 kg·ha^-1^; P_2_O_5–_100 kg·ha^-1^; K_2_O 15 kg·ha^-1^. W_1_ denotes 1650 m^3^·ha^-1^; W_2_ denotes 2500 m^3^·ha^-1^; W_3_ denotes 3350 m^3^·ha^-1^. Uppercase letters denote significant differences (*P* < 0.05) between irrigation rates within the same fertilization regime. Lowercase letters indicate significant differences (*P* < 0.05) between fertilization levels within the same irrigation treatment.

Variance analysis of both cultivars (**Table 3**) revealed significant fertilization × irrigation interactions (*P* < 0.05) for soil TN, TP, and SOC in the 0–20 cm layer. Fertilization independently significantly affected AN and AP across soil depths (*P* < 0.05). Under F_0_ and F_1_ treatments, soil TN, TP, AN, and SOC exhibited initial increases followed by decreases with rising irrigation volume (**Figure 2**). Conversely, F_2_ treatment showed progressive declines in SOC, TN, AN, and TP with increasing irrigation. Maximum nutrient concentrations occurred under F_2_W_1_: SOC: 1.92 g·kg^-1^ (WL354HQ) and 1.25 g·kg^-1^ (Xinmu No.4), TN: 1.82 g·kg^-1^ and 1.98 g·kg^-1^, TP: 17.64 g·kg^-1^ and 13.75 g·kg^-1^, AN: 158.27 mg·kg^-1^ and 150.11 mg·kg^-1^. These values represented significant increases of 22.9%/21.1% (SOC), 66.0%/64.3% (TP), 19.3%/24.6% (TN), and 29.6%/19.4% (AN) over F0W1 (*P* < 0.05). Significant interactions (P < 0.05) persisted for SOC, TN, and TP in the 20–40 cm and 40–60 cm layers. Under F_2_ treatment, SOC and TN decreased progressively with irrigation intensification. At 20–40 cm depth, WL354HQ showed 22.6% higher SOC under F_1_W_2_ (12.84 g·kg^-1^) versus F_1_W_1_ (10.47 g·kg^-1^; *P* < 0.05). The F_2_W_3_ treatment caused 22.6% TN reduction in WL354HQ (0.48 and 0.62 g·kg^-1^) and 34.8% TP decrease in Xinmu No.4 (0.60 and 0.92 g·kg^-1^) relative to F_2_W_1_ at the 40–60 cm depth (*P* < 0.05). Notably, under F_2_ fertilization, AP content in the 0–20 cm layer decreased by 12.59% (WL354HQ) and 5.52% (Xinmu No.4) under high irrigation (W_3_) compared to W_1_ (*P* < 0.05), indicating leaching effects. This demonstrates that medium irrigation (W_2_) enhances topsoil nutrient retention, whereas high irrigation (W_3_) accelerates nutrient leaching to deeper layers.

**Table 3:** ANOVA of SOC, TN, TP, AP and AN contents in alfalfa (WL354HQ and XM No.4), across soil depths under irrigation and fertilization management.

Soil layer	Variation	SOC^1^		TN^2^		TP^3^		AP^4^		AN^5^	
		WL354HQ	XM No.4	WL354HQ	XM No.4	WL354HQ	XM No.4	WL354HQ	XM No.4	WL354HQ	XM No.4
0–20 cm	F^6^	17.99**	139.52**	9.38*	21.10**	39.37**	50.31**	120.52**	49.11**	4.29*	9.62*
	W^7^	9.04*	8.71*	5.31*	4.01*	6.57*	5.18*	3.72ns	5.64*	0.27ns	0.77ns
	F × W^8^	2.71*	0.34ns	13.92**	3.92*	3.02*	2.78*	0.79ns	1.62ns	8.34**	2.13ns
20–40 cm	F	0.82ns	8.63*	0.89ns	3.29ns	0.89ns	4.02ns	49.36**	0.97*	4.66*	6.09*
	W	0.05ns	0.15ns	0.38ns	0.53ns	0.22ns	0.09ns	3.13ns	1.45ns	0.17ns	1.26ns
	F × W	26.66**	9.28**	26.50*	7.58**	55.79**	22.01**	0.31ns	3.01*	1.94ns	6.50ns
40–60 cm	F	1.95ns	0.67ns	0.71ns	1.89ns	32.54**	2.62ns	10.53*	10.91*	8.70*	17.66**
	W	0.12ns	0.16ns	0.01ns	0.14ns	2.22ns	1.20ns	1.41ns	1.53ns	0.38ns	1.28ns
	F × W	18.04**	11.90**	7.49**	7.68**	6.88**	7.39**	0.65ns	0.67ns	7.00**	1.84ns

^1^Soil organic carbon (SOC) content, ^2^total nitrogen (TN) content, ^3^total phosphorus (TP), ^4^available phosphorus (AP), and ^5^alkali-hydrolyzable nitrogen (AN) content. F^6^ denotes the degree of variation in fertilizer levels, W^7^ denotes the degree of variation in Irrigation levels, F × W^8^ denotes the degree of variation in the interaction between fertilizer levels and irrigation levels. ** denotes extremely significant differences *P* < 0.01, * denotes significant differences *P* < 0.05, and ns denotes not significant *P* ≥ 0.05.

### 3.2 Photosynthetic pigment content and photosynthetic performance

**Figure 3:**
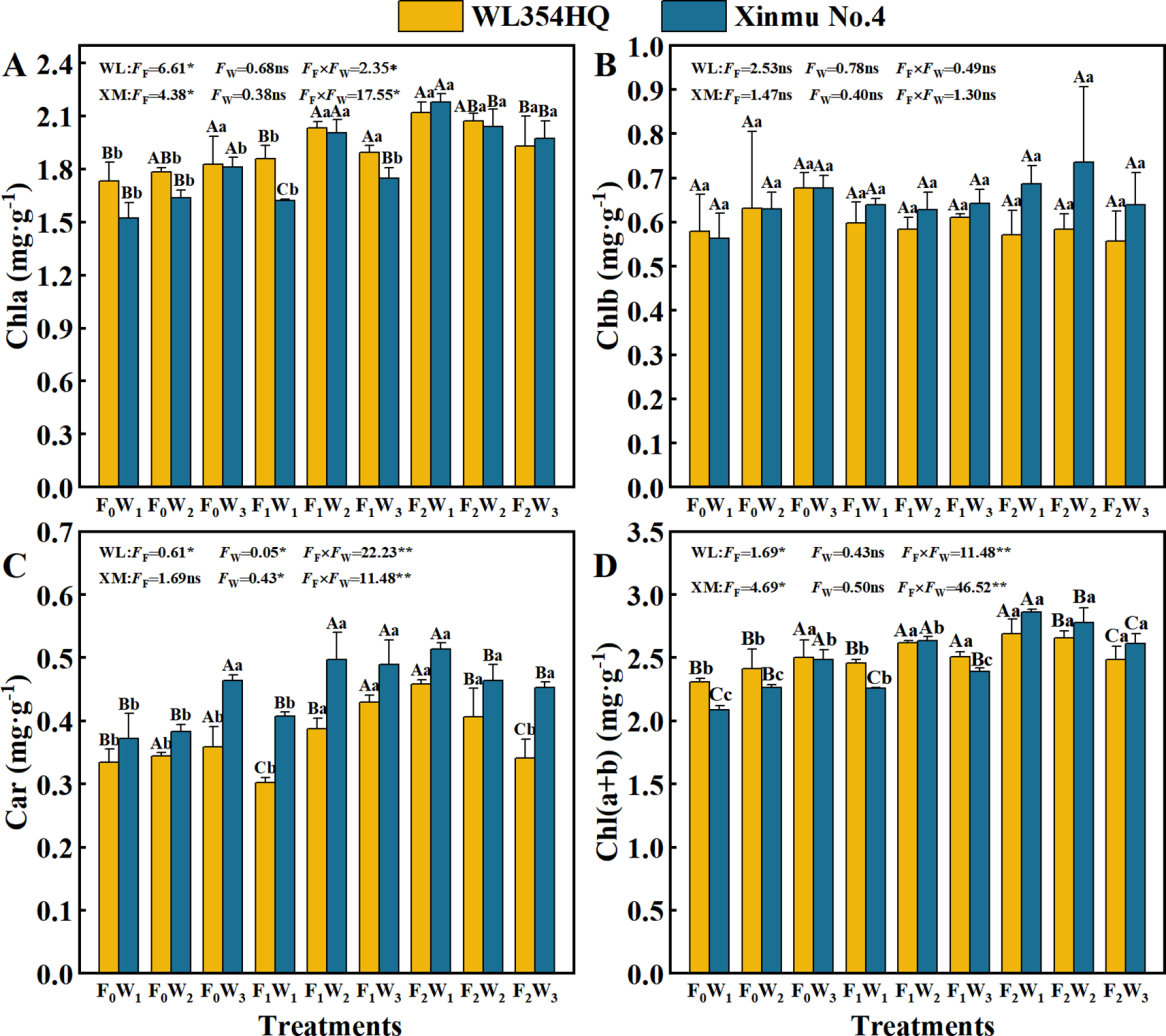
Effects of irrigation-fertilization management on Photosynthetic pigment content and photosynthetic performance in alfalfa (WL354HQ and Xinmu No.4). **(A)** Chla denotes chlorophyll a content, **(B)** Chlb denotes chlorophyll b content, **(C)** Car denotes carotenoid content, **(D)** Ch l(a+b) denotes chlorophyll (a+b) content. Uppercase letters denote significant differences (*P* < 0.05) between irrigation levels within the same fertilization regime. Lowercase letters indicate significant differences (*P* < 0.05) between fertilization levels within the same irrigation treatment. ** denotes *P* < 0.01, * denotes *P* < 0.05, and ns denotes not significant *P* ≥ 0.05. WL denotes WL354HQ, XM denotes Xinmu No.4, *F*_F_ denotes the degree of variation in fertilizer levels, *F*_W_ denotes the degree of variation in Irrigation levels, *F*_F_ × *F*_W_ denotes the degree of variation in the interaction between fertilizer levels and irrigation levels. ** denotes extremely significant differences *P* < 0.01, * denotes significant differences *P* < 0.05, and ns denotes not significant *P* ≥ 0.05.

**Figure 4:**
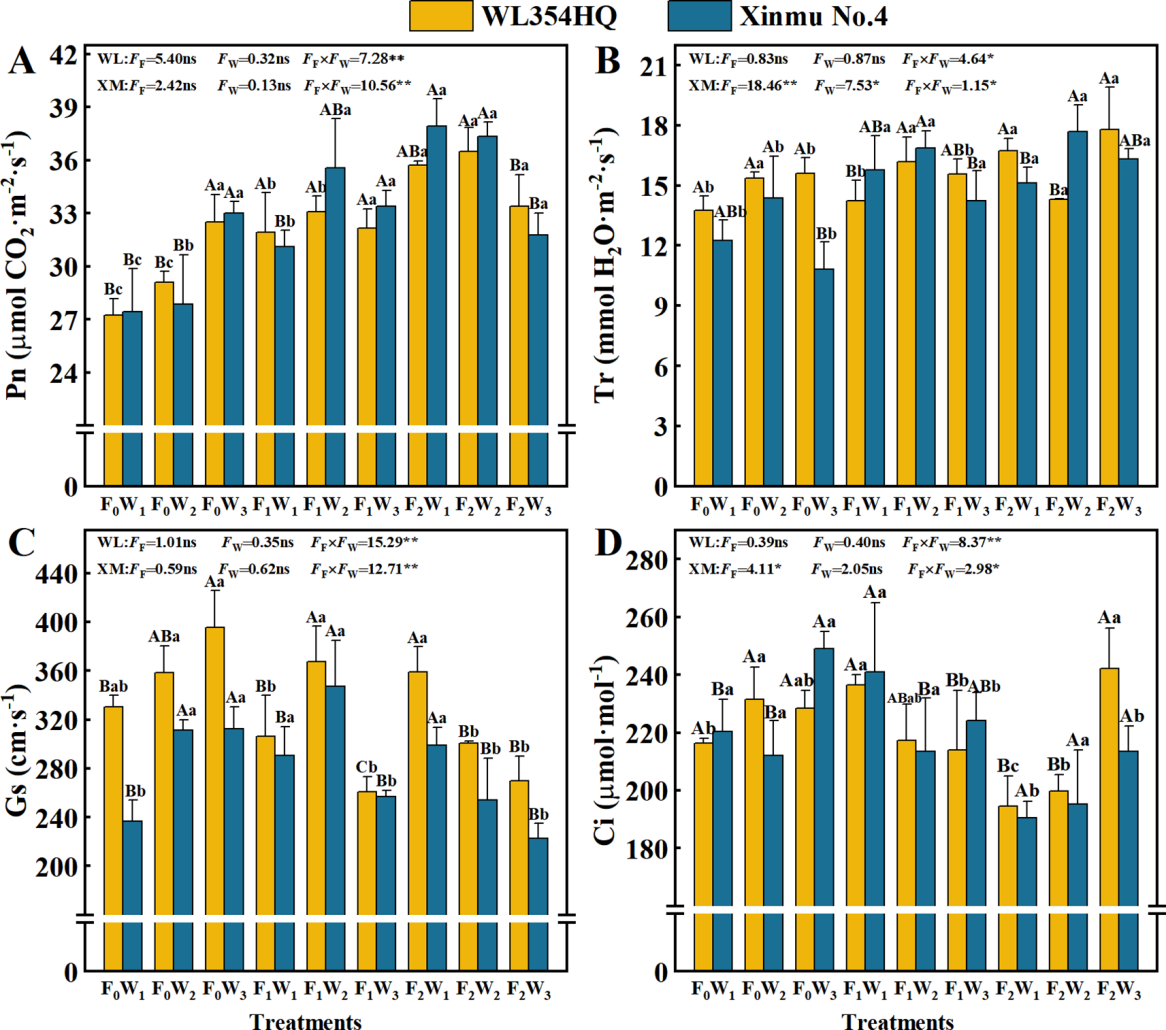
Effects of irrigation-fertilization management on photosynthetic performance in alfalfa (WL354HQ and Xinmu No.4). **(A)** Pn denotes net photosynthetic rate, **(B)** Tr denotes transpiration rate, **(C)** Gs denotes stomatal conductance, **(D)** Ci denotes intercellular CO_2_ concentration. Uppercase letters denote significant differences (*P* < 0.05) between irrigation levels within the same fertilization regime. Lowercase letters indicate significant differences (*P* < 0.05) between fertilization levels within the same irrigation treatment. WL denotes WL354HQ, XM denotes Xinmu No.4, *F*_F_ denotes the degree of variation in fertilizer levels, *F*_W_ denotes the degree of variation in Irrigation levels, *F*_F_ × *F*_W_ denotes the degree of variation in the interaction between fertilizer levels and irrigation levels. ** denotes extremely significant differences *P* < 0.01, * denotes significant differences *P* < 0.05, and ns denotes not significant *P* ≥ 0.05.

Fertilization level and their interactions with irrigation rate significantly (*P* < 0.05) influenced Chl a, Car, and Chl a+b contents in both alfalfa cultivars (**Table 3**; **Figure 3**). The F_2_W_1_ treatment produced highest Chl a contents of 2.12 mg·g^-1^ in WL354HQ and 2.18 mg·g^-1^ in Xinmu No.4, corresponding to 22.5% and 43.4% increases relative to F_0_W_1_, respectively (*P* < 0.05) (**Figure 3A**). WL354HQ exhibited a 39.4% increase in Car content under F_2_W_1_ compared to F_0_W_1_ (**Figure 3C**). Significant (*P* < 0.01) fertilization × irrigation interactions were detected for photosynthetic parameters (**Figure 4**). The F_2_W_2_ treatment achieved maximum Pn (**Figure 4A**) of 36.50 μmol CO_2_·m^-2^·s^-1^ in WL354HQ and 37.32 μmol CO_2_·m^-2^·s^-1^ in Xinmu No.4, representing 34.0% and 36.1% enhancements, respectively, when compared to F_0_W_1_ (*P* < 0.01). The Pn of Xinmu No.4 alfalfa under F_2_W_3_ treatment was 14.9% lower than that under F_2_W_2_ treatment (*P* < 0.05). Tr and Ci showed alfalfa cultivar specificity (**Figures 4B, D**). The Tr of WL354HQ under F_2_W_3_ treatment (17.77 mmol H_2_O·m^-2^·s^-1^) was significantly (*P* < 0.05) higher than that under F_2_W_2_ treatment (14.32 mmol H_2_O·m^-2^·s^-1^), and the Ci increased to 242.11 μmol·mol^-1^ at the same time. The Tr of Xinmu No.4 was the highest under F_2_W_2_ treatment (17.68 mmol H_2_O·m^-2^·s^-1^), but the Ci remained low (195.31 μmol·mol^-1^).

### 3.3 Growth index

**Figure 5:**
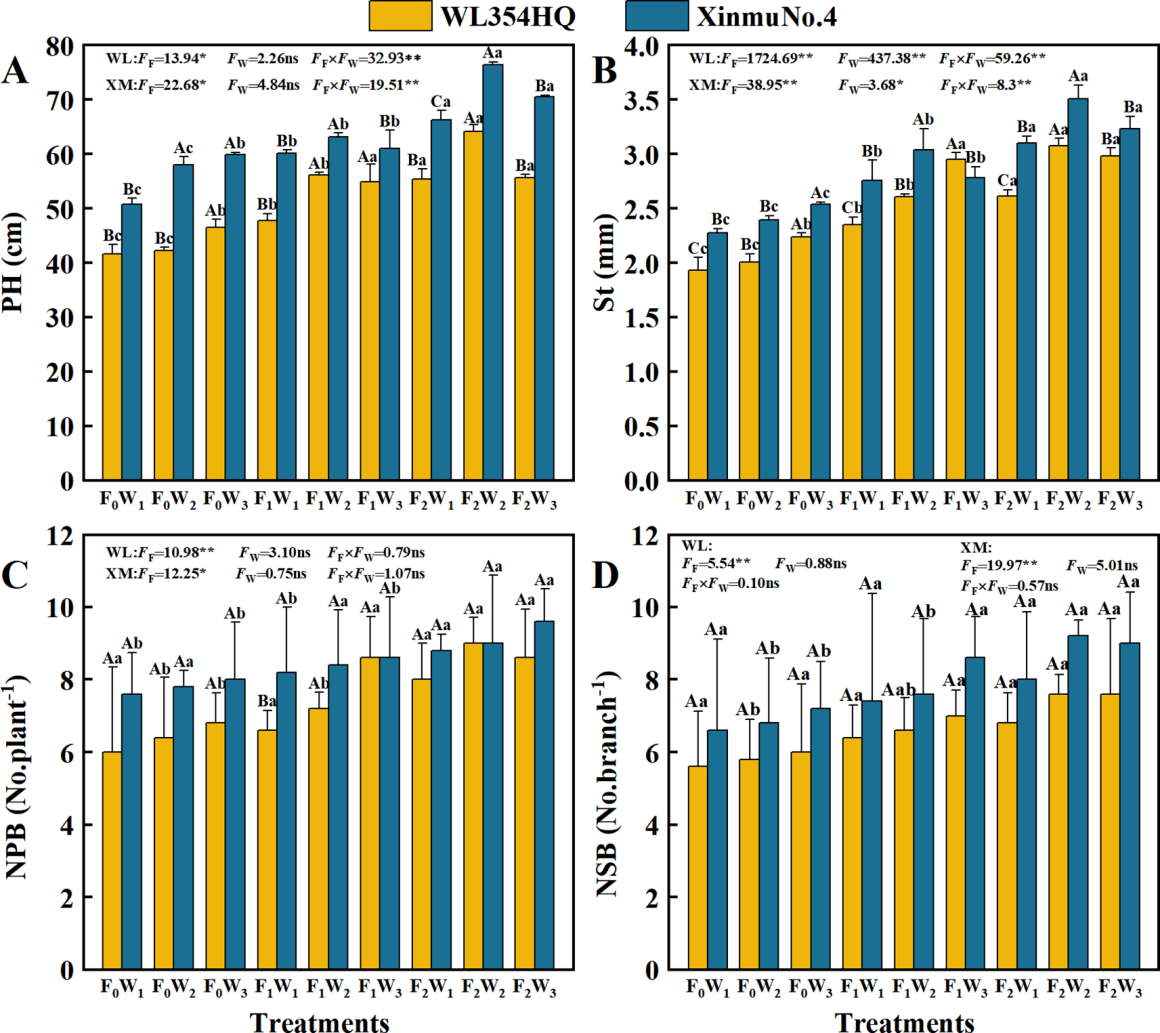
Effects of irrigation-fertilization management on growth index of alfalfa (WL354HQ and Xinmu No.4). **(A)** PH denotes plant height, **(B)** St denotes stem thick, **(C)** NPB denotes number of primary branches, **(D)** NSB denotes number of secondary branches. Uppercase letters denote significant differences (*P* < 0.05) between irrigation levels within the same fertilization regime. Lowercase letters indicate significant differences (*P* < 0.05) between fertilization levels within the same irrigation treatment. WL denotes WL354HQ, XM denotes Xinmu No.4, *F*_F_ denotes the degree of variation in fertilizer levels, *F*_W_ denotes the degree of variation in Irrigation levels, *F*_F_ × *F*_W_ denotes the degree of variation in the interaction between fertilizer levels and irrigation levels. ** denotes extremely significant differences *P* < 0.01, * denotes significant differences *P* < 0.05, and ns denotes not significant *P* ≥ 0.05.

Fertilization level and irrigation rate significantly (*P* < 0.05) affected PH and St in alfalfa (**Figures 5A, B**). The F_2_W_2_ treatment produced maximum PH values of 64.18 cm in WL354HQ and 76.35 cm in Xinmu No.4, representing 54.01% and 50.26% increases, respectively, compared to F_0_W_1_ (41.68 and 50.08 cm) (*P* < 0.05). St showed a fertilization-dependent increase, reaching maxima of 3.07 mm in WL354HQ and 3.51 mm in Xinmu No.4 in the F_2_W_2_ treatment. Both PH and St under F_1_ and F_2_ treatments exhibited quadratic responses to increased irrigation rate. high irrigation (W_3_) under F_2_ fertilization caused 13.3% PH reduction in WL354HQ and 8.0% St decrease in Xinmu No.4 when compared to F_2_W_2_ (*P* < 0.05), demonstrating that supra-optimal irrigation negates fertilizer-enhanced growth. Fertilization level significantly (*P* < 0.05) influenced NPB and NSB (**Figures 5C, D**). NPB showed linear increases with fertilization intensity, with F_2_ treatments producing 33.3-36.8% more branches than F_0_. Peak values of 8.6 No.plant^-1^ (WL354HQ) and 9.6 No.plant^-1^ (Xinmu No.4) were recorded under F_2_W_3_. The main effects of irrigation rates and water-fertilizer interaction effects showed no significant impacts on branch numbers (*P* > 0.05), demonstrating that branch development in alfalfa was predominantly governed by nutrient supply rather than irrigation rate.

### 3.4 The constituent factors of alfalfa seed yield and resource use efficiency

**Figure 6:**
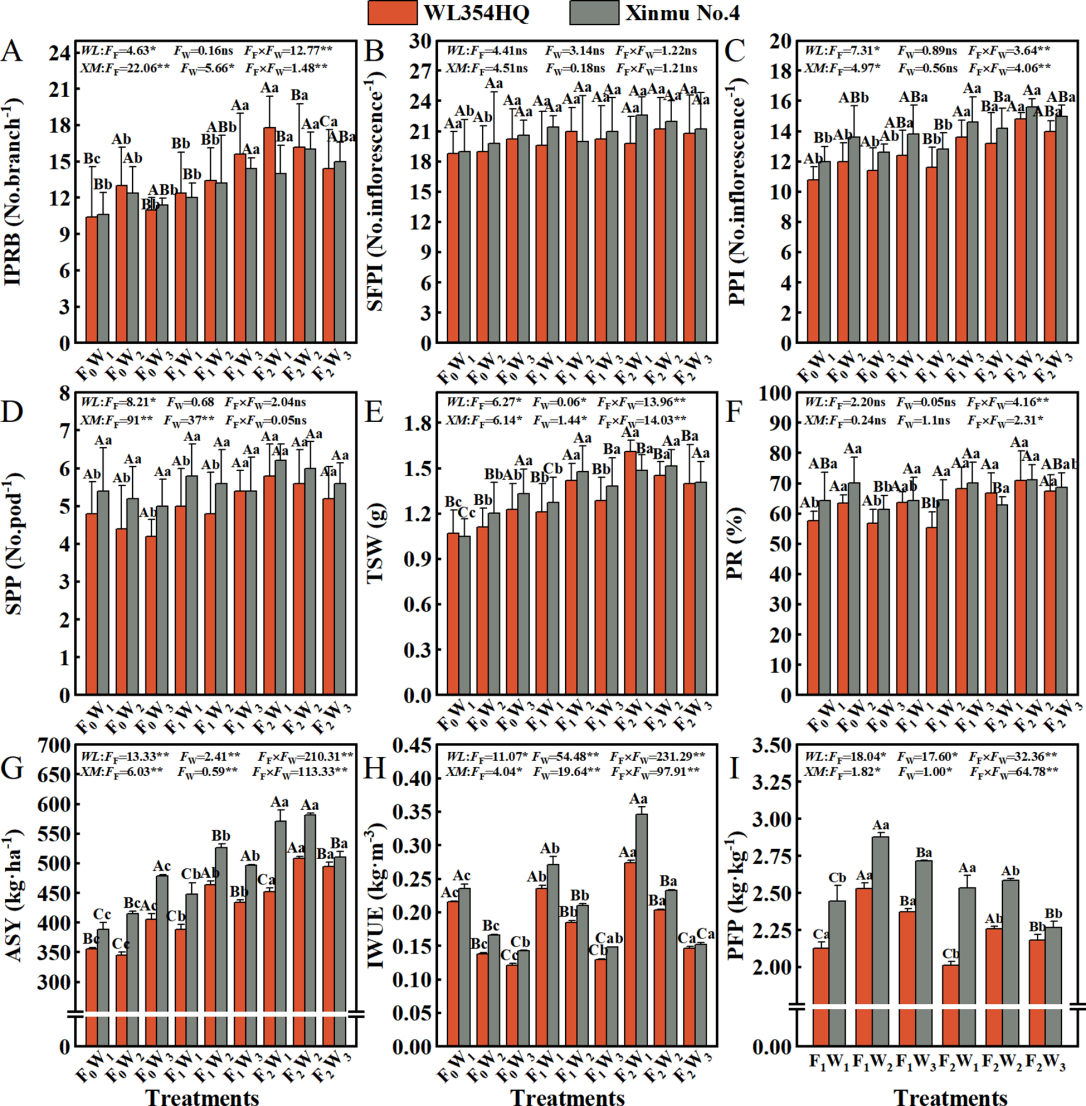
Effects of irrigation-fertilization management on the constituent factors of alfalfa (WL354HQ and Xinmu No.4) seed yield and resource use efficiency. **(A)** IPRB denotes number of inflorescences per reproductive branch, **(B)** SFPI denotes number of small flowers per inflorescence, **(C)** PPI denotes number of pods per inflorescence, **(D)** SPP denotes seeds per pod, **(E)** TSW denotes thousand seed weight, **(F)** PR denotes podding rate, **(G)** ASY denotes actual seed yield, **(H)** IWUE denotes irrigation water use efficiency, and **(I)** PFP denotes partial fertilizer productivity. Uppercase letters denote significant differences (*P* < 0.05) between irrigation levels within the same fertilization regime. Lowercase letters indicate significant differences (*P* < 0.05) between fertilization levels within the same irrigation treatment. WL denotes WL354HQ, XM denotes Xinmu No.4, *F*_F_ denotes the degree of variation in fertilizer levels, *F*_W_ denotes the degree of variation in Irrigation levels, *F*_F_ × *F*_W_ denotes the degree of variation in the interaction between fertilizer levels and irrigation levels. ** denotes extremely significant differences *P* < 0.01, * denotes significant differences *P* < 0.05, and ns denotes not significant *P* ≥ 0.05.

Fertilization × irrigation interactions significantly (*P* < 0.05) regulated yield components including IPRB, PPI, PR, TSW and ASY in both WL354HQ and Xinmu No.4 alfalfa cultivars (**Figure 6**). Progressive fertilization intensity elicited gradual improvements in yield-determining traits across all irrigation rate. The F_2_W_1_ treatment achieved highest IPRB values of 17.8 and 14.0 No.branch^-1^ in WL354HQ and Xinmu No.4, respectively, representing a 71.2% and 32.1% increases over F_0_W_1_ (*P* < 0.01, **Figure 6A**). F_2_W_2_ treatment optimized PPI to 14.8 and 15.6 No.inflorescence^-1^ in WL354HQ and Xinmu No.4, corresponding to 37.0% and 30.0% enhancements (**Figure 6C**), respectively, when compared to F_0_W_1_ (*P* < 0.01). Maximum ASY under F_2_W_2_ reached 508.19 kg·ha^-1^ for WL354HQ and 581.99 kg·ha^-1^ for Xinmu No.4, surpassing F_0_W_1_ by 42.8% and 49.8% (*P* < 0.01). High irrigation (W_3_) under F_2_ fertilization resulted in 12.3% ASY reduction in Xinmu No.4 compared to F_2_W_2_ (*P* < 0.05, **Figure 6G**). Increased fertilization rate from F_0_ to F_2_ increased TSW by 31.56% in WL354HQ (1.13 and 1.48 g) and 23.52% in Xinmu No.4 (1.19 and 1.47 g) (**Figure 6E**). The F_2_W_1_ treatment achieved highest IWUE of 0.27 kg·m^-3^ (WL354HQ) and 0.35 kg·m^-3^ (Xinmu No.4) (**Figure 6H**), demonstrating optimized water productivity under balanced fertilization-irrigation management. The F_1_W_2_ treatment attained maximum PFP of 2.53 and 2.88 kg·kg^-1^, outperforming F_2_W_2_ by 11.9% and 13.9%, repectively (*P* < 0.05, **Figure 6I**).

### 3.5 Quality of seeds

**Figure 7:**
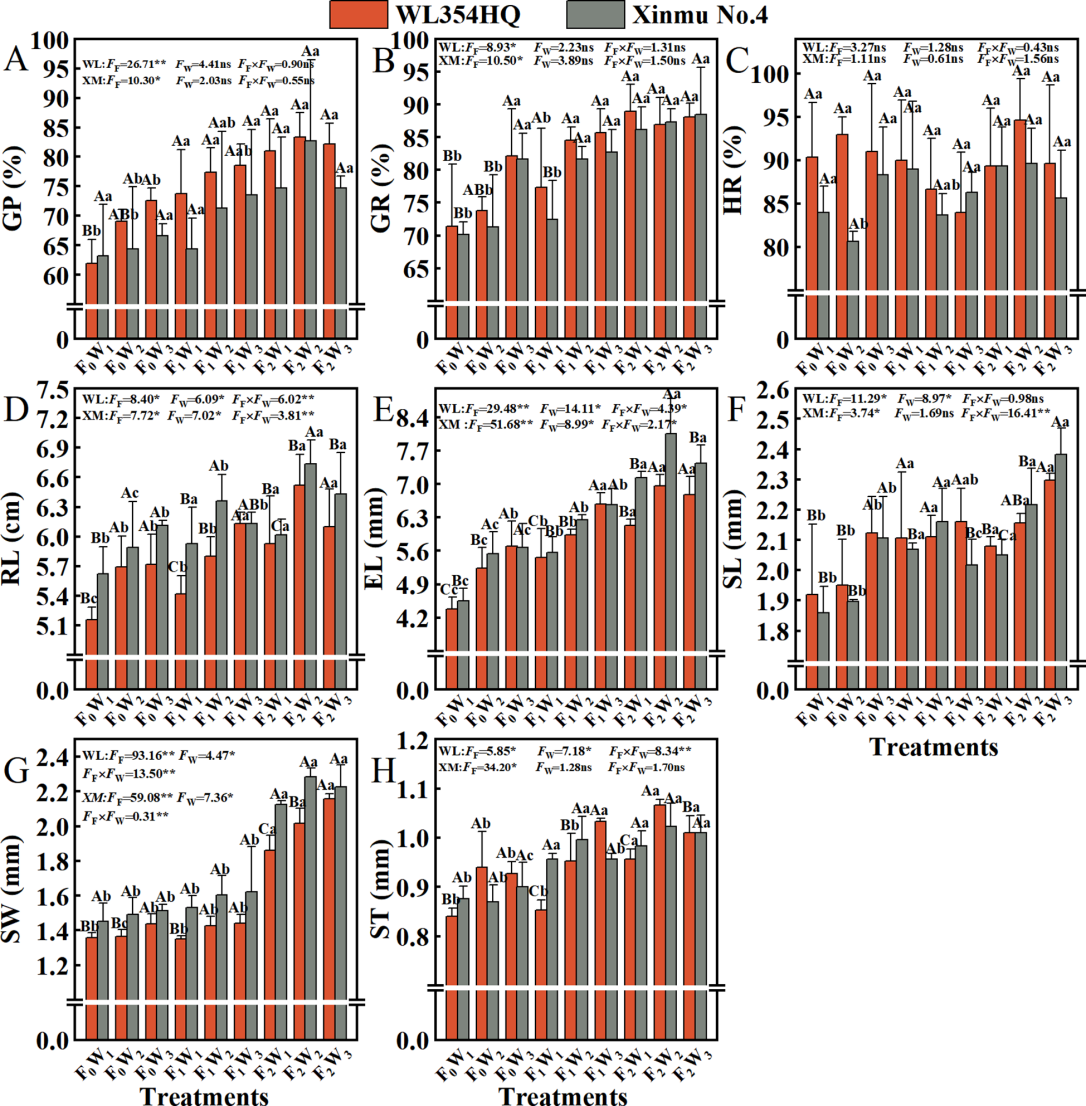
Effects of irrigation-fertilization management on the quality of alfalfa (WL354HQ and Xinmu No.4). **(A)** GP denotes germination potential, **(B)** GR denotes germination rate, **(C)** HR denotes hard seed rate, **(D)** RL denotes radicle length, **(E)** EL denotes embryo length, **(F)** SL denotes seed length, **(G)** SW denotes seed width, **(H)** ST denotes seed thickness. Uppercase letters denote significant differences (*P* < 0.05) between irrigation levels within the same fertilization regime. Lowercase letters indicate significant differences (*P* < 0.05) between fertilization levels within the same irrigation treatment. WL denotes WL354HQ, XM denotes Xinmu No.4, *F*_F_ denotes the degree of variation in fertilizer levels, *F*_W_ denotes the degree of variation in Irrigation levels, *F*_F_ × *F*_W_ denotes the degree of variation in the interaction between fertilizer levels and irrigation levels. ** denotes extremely significant differences *P* < 0.01, * denotes significant differences *P* < 0.05, and ns denotes not significant *P* ≥ 0.05.

Fertilization rate had a significant (*P* < 0.05) effect on GP and GR (**Figure 7**). With the increase of fertilization level F_0_ to F_2_, the GP and GR of WL354HQ and Xinmu No.4 alfalfa showed an increasing trend, and the GP was highest in F_2_W_2_ treatment (0.83% and 0.83%), which were 33.87% and 31.75% higher than that of F_0_W_1_, respectively (*P* < 0.01). It is worth noting that the single factor of irrigation rate had no significant (*P* > 0.05) effect on GR, but the Xinmu No.4 GR under F_0_W_3_ treatment was 17.1% higher than that of F_0_W_1_ (*P* < 0.05), and the mean value was stable at 0.84-0.95%, indicating that water and fertilizer management did not change seed physical dormancy characteristic. Fertilization level, irrigation rate and their interaction had significant (*P* < 0.01) effects on EL and RL (**Figures 7D, E**). The EL (6.52 mm and 6.74 mm) and RL (6.96 cm and 8.05 cm) of WL354HQ and Xinmu No.4 alfalfa under F_2_W_2_ treatment were the highest, which increased by 26.6 and 19.9%, 58.9%, and 76.9%, respectively, when compared with F_0_W_1_ (*P* < 0.05). High irrigation (W_3_) under conventional fertilization (F_2_) resulted in a decrease in RL. The RL of Xinmu No.4 under F_2_W_3_ treatment was 7.7% lower than that under F_2_W_2_ treatment (*P* < 0.05). Among the seed morphological indexes, SW was significantly (*P* < 0.01) affected by fertilization level (**Figure 7G**), irrigation rate and their interaction. WL354HQ and Xinmu No.4 alfalfa had the largest SW (2.02 mm and 2.28 mm) under F_2_W_2_ treatment, which was 48.5% and 57.2% higher than F_0_W_1_, respectively (*P* < 0.01). It is worth noting that the ST (**Figure 7H**) of WL354HQ was significantly (*P* < 0.05) higher in F_1_W_3_ treatment (1.03 mm) than in F_1_W_1_ treatment (0.85 mm).

### 3.6 Correlation analysis

**Figure 8:**
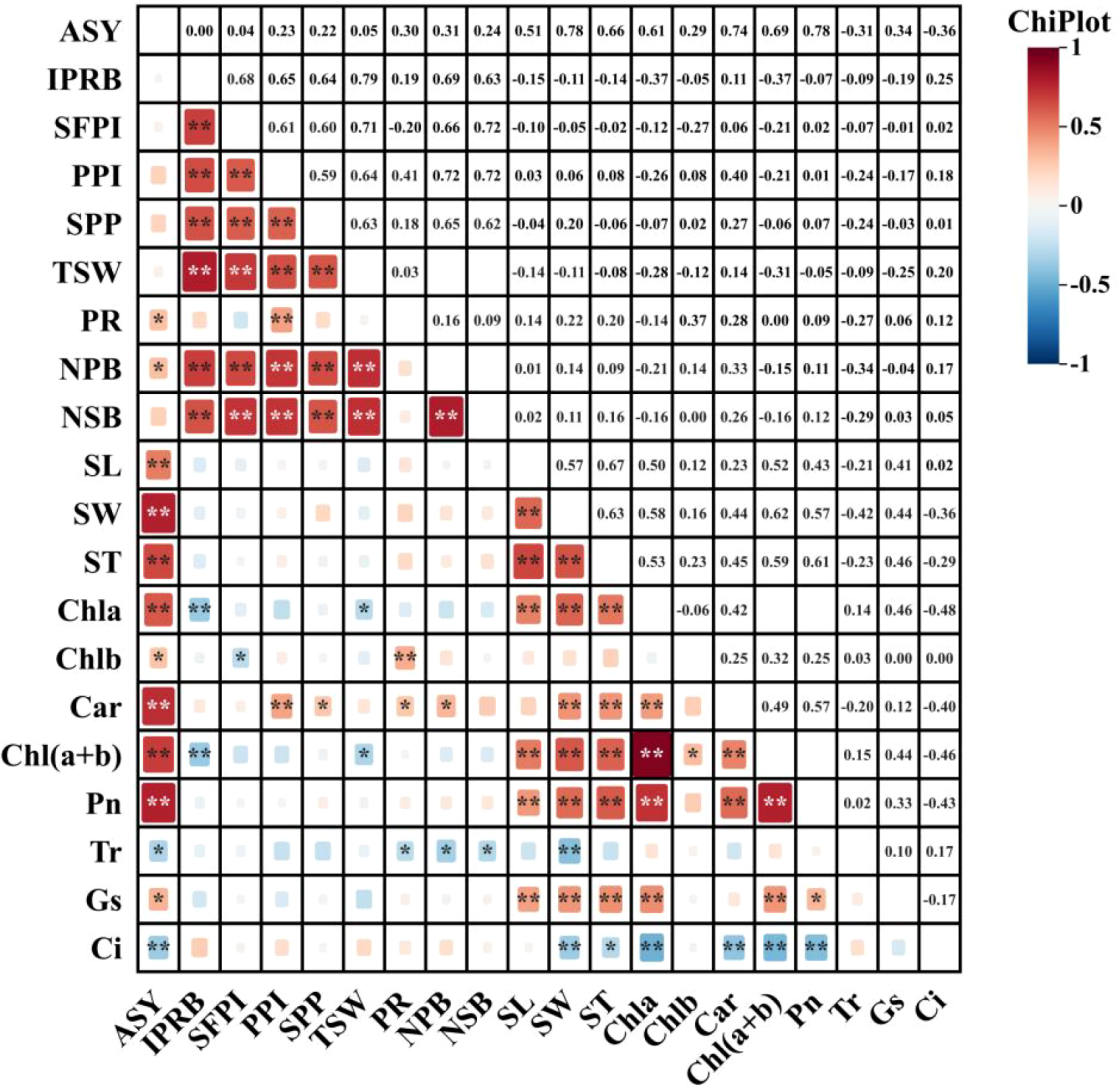
Pearson correlation analysis showed the relationship between alfalfa (WL354HQ and Xinmu No.4) seed composition factors, photosynthetic performance and yield. The size of the squares shows a significant level, and the color of the squares shows a positive correlation or a negative correlation. *P* values less than 0.01 and 0.05 are indicated by asterisks and dot symbols “**”, and “*”. The abbreviations used in the figure are: ASY denotes actual seed yield, IPRB denotes number of inflorescences per reproductive branch, SFPI denotes number of small flowers per inflorescence, PPI denotes number of pods per inflorescence, SPP denotes seeds per pod, TSW denotes thousand seed weight, PR denotes podding rate, NPB denotes number of primary branches, NSB denotes number of secondary branches, SL denotes seed length, SW denotes seed width, ST denotes seed thickness, Chla denotes chlorophyll a content, Chlb denotes chlorophyll b content, Car denotes carotenoid content, Ch l(a+b) denotes chlorophyll (a+b) content, Pn denotes net photosynthetic rate, Tr denotes transpiration rate, Gs denotes stomatal conductance, and Ci denotes intercellular CO_2_ concentration.

**Figure 9:**
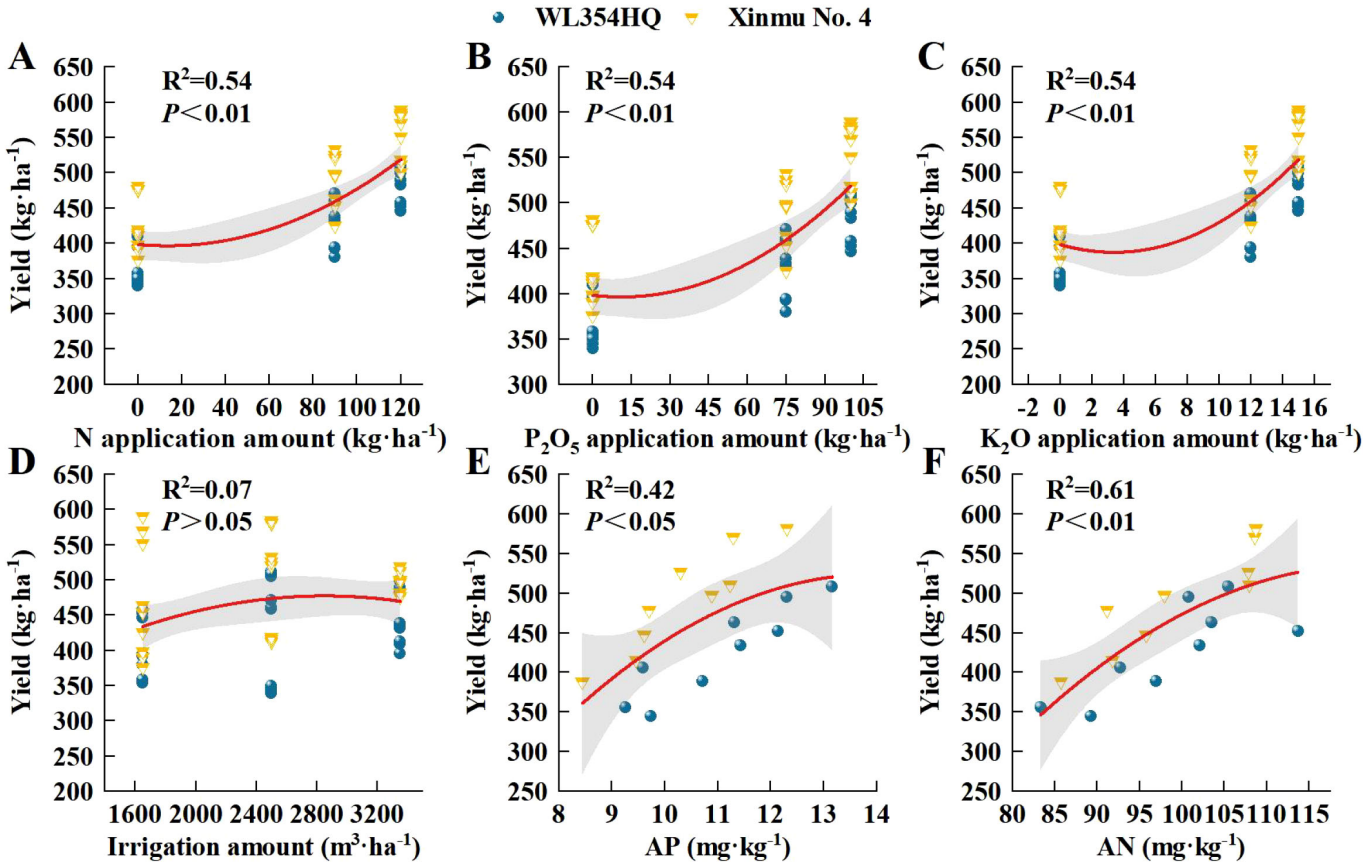
Analysis of the influence of irrigation-fertilization management on alfalfa (WL354HQ and Xinmu No.4) seed yield and its correlation with AP and AN contents. **(A–D)** represents the relationship between alfalfa seed yield and the input amounts of N, P, and K as well as the irrigation volume. **(E, F)** represents the relationship between alfalfa seed yield and soil AP and AN contents. Polynomial fitting determines that the error zone (shaded area) corresponds to the 95% confidence interval of the relationship, and the red line represents the linear trend line. The abbreviations used in the picture are: Yield denotes seed yield, AP denotes available phosphorus contents, AN denotes alkali-hydrolyzable nitrogen contents.

**Figure 10:**
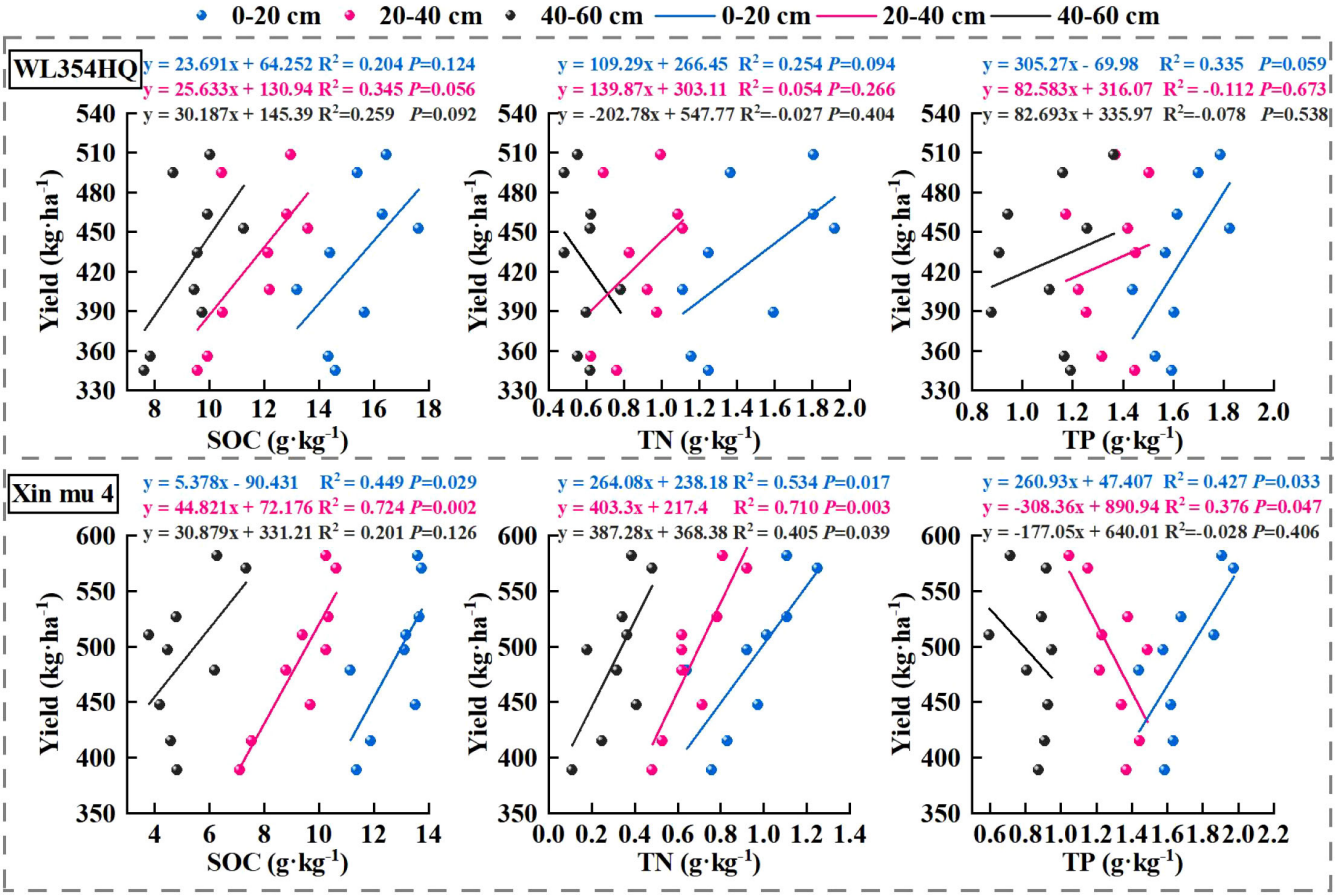
The correlation between alfalfa (WL354HQ and Xinmu No.4) seed yield and the contents of soil organic carbon (SOC), total nitrogen (TN) and total phosphorus (TP) at different soil depths.

**Figure 11:**
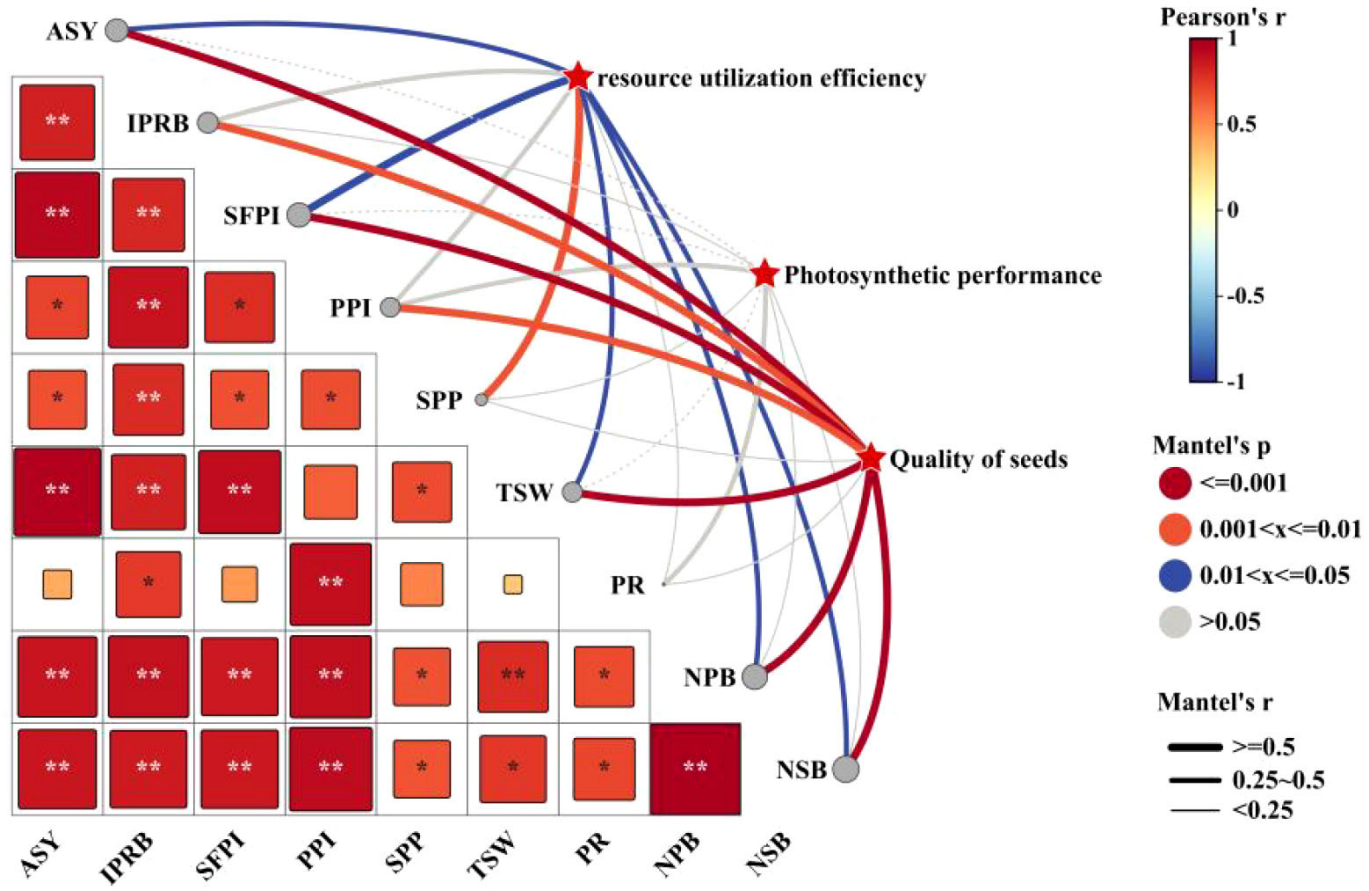
The correlation analysis of Mantel test shows the relationship between alfalfa seed quality, resource use efficiency and photosynthetic performance. The thickness of the line shows the R^2^ level, the size of the square shows the significant level, and the color of the square shows a positive correlation or a negative correlation. *P* values less than 0.01 and 0.05 are indicated by asterisks and dot symbols “**”, and “*”. The abbreviations used in the picture are: ASY denotes actual seed yield, IPRB denotes number of inflorescences per reproductive branch, SFPI denotes number of small flowers per inflorescence, PPI denotes number of pods per inflorescence, SPP denotes seeds per pod, TSW denotes thousand seed weight, PR denotes podding rate, NPB denotes number of primary branches, and NSB denotes number of secondary branches.

In order to further analyze the relationship between alfalfa seed composition, photosynthetic performance, SOC, TN, and TP content at the different soil depths and seed yield. Pearson correlation analysis, mantel test and linear fitting were performed on these indicators. The seed yield of alfalfa was significantly positively correlated with SL, SW, ST, Chl a, Car, Chl (a+b) content and Pn (*P* < 0.01), positively correlated with PR, NPB, and Tr (*P* < 0.05), negatively correlated with Gs and Ci (*P* < 0.05) (**Figure 8**), At the 0–20 cm soil layer, seed yield showed significant positive linear relationships with TN (R^2^ = 0.254 and 0.534) and TP (R^2^ = 0.335 and 0.427), Within the 20–40 cm depth, stronger correlations were detected between ASY and SOC (R^2^ = 0.354 and 0.724) and TN (R^2^ = 0.054 and 0.710), Within the 0–60 cm depth, stronger correlations were detected between ASY and AP (R^2^ = 0.42, *P* < 0.05) and AN (R^2^ = 0.61, *P* < 0.01) (**Figure 9**), respectively, which were positively correlated (but not significant; *P* > 0.05) with SOC and TN content at the 40–60 cm soil depth (**Figure 10**). There was a significant (*P* < 0.01) positive correlation between seed yield components and seed quality, and a significant (*P* < 0.05) positive correlation with resource use efficiency (**Figure 11**), The ASH was significantly positively correlated with the input of NPK (R^2^ = 0.54, *P* < 0.01) (**Figure 9**).

### 3.7 Principal component analysis and comprehensive evaluation

**Figure 12:**
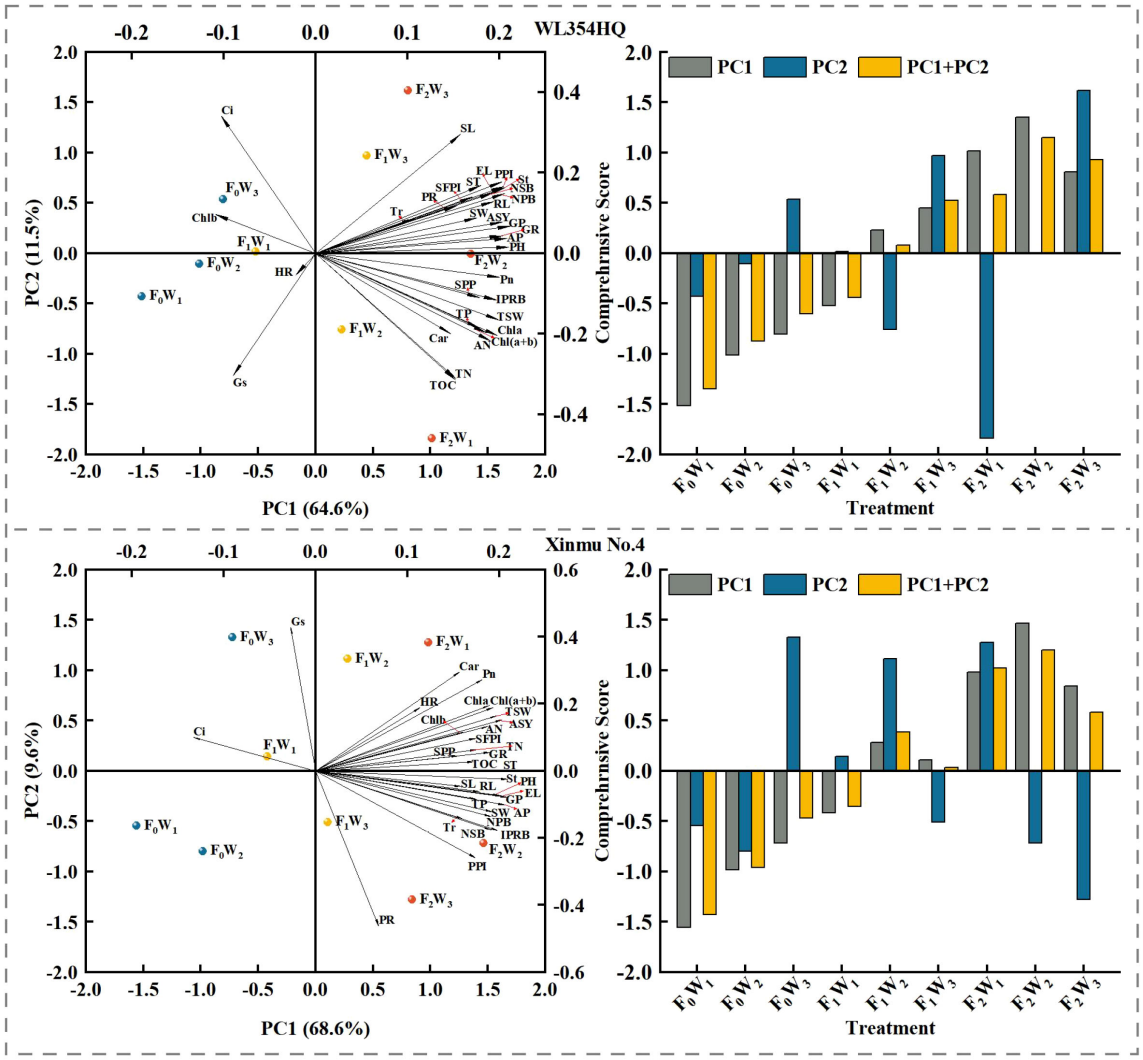
Principal component analysis and comprehensive score of the measured indicators under irrigation-fertilization management. The abbreviations used in the picture are: SOC denotes soil organic carbon content, TN denotes total nitrogen content, TP denotes total phosphorus content, AP denotes available phosphorus contents, AN denotes alkali-hydrolyzable nitrogen contents, Chla denotes chlorophyll a content, Chlb denotes chlorophyll b content, Car denotes carotenoid content, Ch l(a+b) denotes chlorophyll (a+b) content, Pn denotes net photosynthetic rate, Tr denotes transpiration rate, Gs denotes stomatal conductance, Ci denotes intercellular CO_2_ concentration, PH denotes plant height, St denotes stem thick, NPB denotes number of primary branches, NSB denotes number of secondary branches, IPRB denotes number of inflorescences per reproductive branch, SFPI denotes number of small flowers per inflorescence, PPI denotes number of pods per inflorescence, SPP denotes seeds per pod, TSW denotes thousand seed weight, PR denotes podding rate, ASY denotes actual seed yield, IWUE denotes irrigation water use efficiency, PFP denotes partial fertilizer productivity, GP denotes germination potential, GR denotes germination rate, HR denotes hard seed rate, RL denotes radicle length, EL denotes embryo length, SL denotes seed length, SW denotes seed width, ST denotes seed thickness.

Principal component analysis was performed on 29 indicators, including seed composition factors, seed quality, photosynthetic performance, and soil nutrient content of the two cultivars of alfalfa. for both cases (WL354HQ and Xinmu No.4), PC1 is related to fertilizer addition and soil water availability (irrigation). On the left extreme of PC1, F0 is located (less fertility), F_1_ in the meddle, and F_2_ on the right (more fertility). The three water availability levels were located within each level of fertilizer addition, with W_1_ consistently located more to the left than W_2_ and W_3_. For WL354HQ the differences between the treatments shown by PC1 accounted for 64.6%, and 68.6% for Xinmu No.4. The contrasts shown by PC1 on the graphs indicate that for WL354HQ, Ci, Chlb, and Gs increase with F_0_ while the other variables decrease; meanwhile, with irrigation increased, the values of these 3 variables decreased and all the other variables increased, but HR. For both cases, PC2 was mostly explained by the water availability (irrigation), then, for both cultivars, in general, the increasing water availability moved from negative PC2 towards positive PC2, accounting for 11.6% of the differences between the treatments for WL354HQ, and 9.6% for Xinmu No.4. WL354HQ showed a strong contrast, which consisted on increasing Ci and SL with increasing irrigation, at the same time that Gs, TOC and TN decreased, with decreasing irrigation, the contrary happened. Xinmu No.4 behaved differently, with increasing irrigation Gs, Car, and Pn increased, and PR and PPI decreased; the inverse occurred with decreasing irrigation (**Figure 12**).

## 4 Discussion

### 4.1 Effects of irrigation-fertilization management on soil nutrients in alfalfa

Soil nutrients serve as fundamental drivers of crop growth and development. Water and fertilizer management optimizes the rhizosphere microenvironment, thereby enhancing the effectiveness of soil nutrients on plant growth (Acharya et al., 2019). This study showed that fertilization level and irrigation rate had significant regulatory effects on the vertical distribution of soil nutrients in alfalfa. In the 0–20 cm layer, high irrigation (W_3_) significantly reduced SOC and TN content by 24.5% and 18.7% respectively, compared to moderate irrigation (W_1_/W_2_). This reduction is primarily attributed to nutrient leaching under high water flux (Yi et al., 2022). When high irrigation occurs, the proportion of soil macropore flow increases, resulting in a 2.7-fold increase in the migration rate of nitrate N and available P to the 40–60 cm soil layer (Wu et al., 2023). In the 0–20 cm soil depth, the 24.51% rise in available N and 37.54% in available P under F_2_W_2_, in addition this phenomenon is consistent with the research results of predecessors, moderate irrigation improves soil porosity and water/nutrient retention capacity, primarily through inducing a 47.3% increase in shallow root biomass (0–30 cm) and root exudate secretion (Liu et al., 2018). However, high irrigation destabilizes soil aggregates, accelerating nutrient leaching – particularly mobile NO_3_^–^N and available P (Zhang and Guo, 2025). In this study, no fertilization treatment, the contents of SOC and TN in the 20–60 cm soil depth showed an opposite trend: W_3_ > W_2_ and W_1_, and this might be due to the fact that in the absence of exogenous addition, water drives the migration of soil nutrients to the deep soil (Cao et al., 2021). There are significant differences in the total nitrogen content of the topsoil among cultivars, which may be due to the specific genotypes of the cultivars (M’Sehli et al., 2008) rather than experimental errors. In addition, in that the abundance of rhizobia in the soil at depth (> 40 cm) (4.2 × 10^5^·g^-1^) was significantly higher than that in shallow soil (1.8 × 10^5^·g^-1^), under high irrigation, the rhizobium abundance was higher and enhancing biological N fixation (Yang et al., 2024). In this study, under F_0_ and F_1_ treatments, at 0–20 cm soil depth soil nutrients showed a single peak distribution with increased irrigation rate, and was highest in the W_2_ treatment. We suggest that this reflects a the mutual feedback effect of water and root (Wu et al., 2025). In that, studies have shown that moderate irrigation can increase the content of available P and ammonium N in surface soil by inducing 47.3% increase of alfalfa root biomass in the upper portion of the soil profile (0–30 cm)and promoting the secretion of citric acid and malic acid (Graham et al., 2022). While fertilizer inputs directly controlled yield (**Figure 9**), the strong AN-yield linkage (R²=0.61) indicates that sustained nutrient bioavailability until harvest is critical for arid-region alfalfa. This aligns with models where optimal irrigation reduces leaching, preserving nutrients for reproductive stages (Gong et al., 2025).

### 4.2 Effects of irrigation-fertilization management on photosynthetic performance in alfalfa

Optimized irrigation and fertilization management improves the planting microenvironment (temperature and humidity), enhances soil nutrient availability, and increases photosynthetic efficiency and pigment accumulation (Rahman et al., 2025). In this study, the chlorophyll a and total chlorophyll content under F_0_ fertilization treatment increased linearly with irrigation volume, and the average values of the indicators, respectively. We propose this reflects a water compensation effect under high evaporative stress in arid regions, where increased water supply elevated leaf relative water content by 14%, effectively mitigating evaporation-induced stress (Boren et al., 2024). Under F_1_ fertilization, chlorophyll a and total chlorophyll exhibited a parabolic response, initially increasing then decreasing with irrigation volume. Below the irrigation threshold, concurrent increases in water, nitrogen (N), phosphorus (P), and potassium (K) inputs: Activated Rubisco activase (RCA activity increased by 42.7%), enhancing carboxylation efficiency and CO_2_ fixation (Lu et al., 2019); Increased PEP carboxylase phosphorylation efficiency (PEPcase activity increased by 28.3%); Strengthened non-photosynthetic limitation advantages through optimized mesophyll conductance (Lollato et al., 2019); Stabilized the oxygen-evolving complex (OEC) of PSII; Increased chloroplast thylakoid stacking density, shortening CO_2_ diffusion distance (Sun et al., 2022), These mechanisms collectively increased total photosynthetic pigments. Above the irrigation threshold: Antioxidant enzyme activities (SOD, POD) decreased significantly, inducing reactive oxygen species (ROS) accumulation and photosynthetic pigment degradation (Noor et al., 2023) characterized by greater chlorophyll a decline than chlorophyll b and disrupted carotenoid-to-chlorophyll ratios. Excessive irrigation increased soil solution permeability and ion toxicity from fertilization, compromising thylakoid membrane integrity (Wei et al., 2024), this may impair root development and heighten water stress sensitivity. Principal component analysis revealed that under identical fertilization, the W_1_ irrigation level had the lowest composite score, with significantly reduced net photosynthetic rate, transpiration rate, and stomatal conductance.We posit this drought condition promotes plant lignification and increases CO_2_ diffusion resistance (Yousfi et al., 2016). Under high irrigation (W_3_), increased biomass accumulation (plant height, stem diameter) accelerated canopy closure (Crookston et al., 2025), elevating respiration and intercellular CO_2_ concentration, and decreasing net photosynthetic rate (Tang et al., 2022). Notably, photosynthetic pigments and net photosynthetic rate were significantly positively correlated with seed yield (*P* < 0.05). Light energy captured by pigments drives carbon assimilation. Resulting assimilates stimulate cytokinin (CTK) accumulation in pod cells, upregulating sucrose transporter SUT2 expression to accelerate sucrose transport to developing seeds (Lu et al., 2019). Concurrently, enhanced auxin polar transport to reproductive organs activates vacuolar acid invertase (vacINV) activity by 38% (*P* < 0.01), promoting sucrose hydrolysis into hexoses that directly supply embryo cell division and storage compound synthesis (Liang et al., 2024). These findings demonstrate that irrigation volume requires dynamic adjustment based on fertilization level. Under low fertilization, increased irrigation can activate photosynthetic potential, whereas high fertilization necessitates controlled irrigation to prevent metabolic imbalance.

### 4.3 Effects of irrigation-fertilization management on growth index and the constituent factors of alfalfa seed yield

Plant growth parameters underpin high crop yields, while seed yield components directly determine final productivity (Wang et al., 2024). Our findings revealed significant (*P* < 0.01) interactive effects of fertilization and irrigation levels on plant height, stem diameter, exceeding F_0_W_1_ by 53.8% and 50.3% for plant height and 58.5% and 60.3% for stem diameter, respectively (*P* < 0.01). These morphological improvements align with physiological mechanisms whereby nitrogen (N) promotes cell elongation, while phosphorus (P) and potassium (K) synergistically enhance vascular tissue development (Wang et al., 2025). Conversely, high irrigation (W_3_) combined with F_2_ fertilization reduced plant height by 9.4% and stem diameter by 13.3%, likely due to hypoxia-induced suppression of root cytochrome oxidase activity under reduced oxygen availability (Wei et al., 2025). Branch development was predominantly regulated by fertilization level (*P* < 0.01). The number of primary branches under F_2_ treatment increased by 33.3–36.8% compared to F_0_, indicating that elevated phosphorus promotes axillary bud differentiation through enhanced meristematic activity (Karamanos et al., 2009). Irrigation alone had no significant effect (*P* > 0.05) on branch number, demonstrating that branch formation depends more critically on nutrient supply than water status. This observation aligns with the priority response of carbon partitioning to N and P signaling in legumes (Lei et al., 2022). Precision irrigation management increased seed yield by 53.9% and nitrogen use efficiency by 26.3% compared to control group (Zhu et al., 2025). The mechanism of increasing yield reflects the synergistic increase of branch number, inflorescence number, and seed number per pod, which in turn improve seed yield (Li et al., 2023b). In the current study, an irrigation volume of 2500 m^3^·ha^-1^ maximized water use efficiency while improving fertilizer partial productivity. This finding is consistent with research in China’s Yellow River irrigation area, where subsurface drip irrigation at 520 mm increased inflorescences and pods per plant, boosting seed yield. This regime also enhanced soil moisture content and root density in the 60–100 cm layer, facilitating deep soil water extraction and improving water use efficiency (Ma et al., 2025). Notably, the F_2_W_2_ treatment achieved a seed yield of 545 kg·ha^-1^. This exceeds the 500 kg·ha^-1^ yield under equivalent drip irrigation (520 mm) in the Yellow River region (Ma et al., 2025) but is lower than the 691 kg·ha^-1^ recorded in Hexi Corridor trials using supplementary irrigation (150 mm during growth) and higher P inputs (225 kg·ha^-1^ diammonium phosphate) (Chen et al., 2025). This comparison demonstrates that in arid regions, moderate irrigation volumes combined with balanced N-P-K fertilization can achieve optimal seed yields with improved resource efficiency.

### 4.4 Effects of irrigation-fertilization management on quality of seeds in alfalfa

Seed morphological traits serve as fundamental indicators of germination capacity and overall seed quality (Ou et al., 2021). Our germination assays revealed that under identical irrigation regimes, the F_2_ fertilization treatment exhibited superior germination potential, radicle length, and seed width compared to F_0_. Furthermore, the response of different fertilization rates to water availability showed different characteristics. In that, F_0_ and F_1_ treatments, germination potential, embryo length and seed width increased linearly with the increased irrigation rate. However, the F_2_ treatment group showed a single peak response. When the irrigation rate exceeded 2500 m^3^·ha^-1^, the above indexes decreased. Balanced N-P-K fertilization enhances thousand-seed weight, morphological indices, soluble sugar, and protein content in seeds (Ran et al., 2024). This management promotes gibberellin (GA), indoleacetic acid (IAA), and abscisic acid (ABA) accumulation in seeds (Xie et al., 2023), thereby improving germination rate, germination potential, and seed vigor to achieve synergistic yield-quality optimization. Consistent with these findings, optimal fertilization significantly improved seed quality in this study, but excessive irrigation (> 2500 m^3^·ha^-1^) negated these benefits (Zhu et al., 2024). Comparative analysis (**Figure 12**) indicates fertilization management exerts greater agronomic impact than irrigation regulation in alfalfa seed production systems. Therefore, balanced water-fertilizer management (e.g., F_2_W_2_) may serve as a regional benchmark for optimizing alfalfa seed quality.

## 5 Conclusion

This study demonstrated that the F_2_W_2_ regime — combining nitrogen (N, 120 kg·ha^-1^), phosphorus (P_2_O_5_, 100 kg·ha^-1^), and potassium (K_2_O, 15 kg·ha^-1^) fertilization with 2500 m^3^·ha^-1^ irrigation — significantly enhances alfalfa seed yield. Specifically, this management: (1) Enriched 0–20 cm soil nutrients, increasing available phosphorus by 37.54% and alkali-hydrolyzable nitrogen by 17.26%; (2) Improved seed quality parameters including thousand-grain weight and germination characteristics; (3) Enhanced photosynthetic performance through elevated pigment accumulation and net photosynthetic rate; (4) Increased branching capacity. Statistical analyses confirmed significant positive correlations (*P* < 0.05) among leaf photosynthetic pigments, net photosynthetic rate, seed morphological traits, 0–20 cm soil total N/P content, and seed yield. In conclusion, the F_2_W_2_ regime provides a scientific foundation for high-yield, high-quality alfalfa cultivation and promotes sustainable seed production systems.

## Data Availability

The original contributions presented in the study are included in the article/supplementary material. Further inquiries can be directed to the corresponding author.
